# Cartilage progenitor cells derived extracellular vesicles-based cell-free strategy for osteoarthritis treatment by efficient inflammation inhibition and extracellular matrix homeostasis restoration

**DOI:** 10.1186/s12951-024-02632-z

**Published:** 2024-06-19

**Authors:** Kai Feng, Feng Wang, Hongfang Chen, Rui Zhang, Jiashuo Liu, Xiaodong Li, Xuetao Xie, Qinglin Kang

**Affiliations:** 1 Department of Orthopedic Surgery, Shanghai Sixth People’s Hospital, Shanghai Jiao Tong University School of Medicine, Shanghai, 200233 China; 2 Department of Orthopaedics, Shanghai Tenth People’s Hospital, Tongji University, Shanghai, 200072 China; 3 Shanghai Key Laboratory of Orthopedic Implants, Department of Orthopedics, Ninth People’s Hospital, Shanghai Jiao Tong University School of Medicine, Shanghai, 200011 China

**Keywords:** Osteoarthritis, Cartilage progenitor cells, Extracellular vesicles, Engineering modification, Inflammation

## Abstract

Osteoarthritis (OA) is a common degenerative joint disease which currently lacks of effective agents. It is therefore urgent and necessary to seek an effective approach that can inhibit inflammation and promote cartilage matrix homeostasis. Cartilage progenitor cells (CPCs) are identified as a cell population of superficial zone in articular cartilage which possess strong migration ability, proliferative capacity, and chondrogenic potential. Recently, the application of CPCs may represent a novel cell therapy strategy for OA treatment. There is growing evidence that extracellular vesicles (EVs) are primary mediators of the benefits of stem cell-based therapy. In this study, we explored the protective effects of CPCs-derived EVs (CPCs-EVs) on IL-1β-induced chondrocytes. We found CPCs-EVs exhibited chondro-protective effects in vitro. Furthermore, our study demonstrated that CPCs-EVs promoted matrix anabolism and inhibited inflammatory response at least partially via blocking STAT3 activation. In addition, liquid chromatography-tandem mass spectrometry analysis identified 991 proteins encapsulated in CPCs-EVs. By bioinformatics analysis, we showed that STAT3 regulatory proteins were enriched in CPCs-EVs and could be transported to chondrocytes. To promoting the protective function of CPCs-EVs in vivo, CPCs-EVs were modified with cationic peptide ε-polylysine-polyethylene-distearyl phosphatidylethanolamine (PPD) for surface charge reverse. In posttraumatic OA mice, our results showed PPD modified CPCs-EVs (PPD-EVs) effectively inhibited extracellular matrix catabolism and attenuated cartilage degeneration. Moreover, PPD-EVs down-regulated inflammatory factors expressions and reduced OA-related pain in OA mice. In ex-vivo cultured OA cartilage explants, PPD-EVs successfully promoted matrix anabolism and inhibited inflammation. Collectively, CPCs-EVs-based cell-free therapy is a promising strategy for OA treatment.

**Supplementary Information:**

The online version contains supplementary material available at 10.1186/s12951-024-02632-z.

## Introduction

Osteoarthritis (OA) is a chronic and progressive joint disease, is primarily characterized by cartilage degradation, subchondral bone remodeling [1], infrapatellar fat pad and synovial membrane inflammation and fibrosis [2, 3], and meniscal degeneration [4]. Cartilage degeneration prevents the joint from withstanding normal biomechanical load and leads to impaired joint function [5], resulting in OA-related pain, disability, and harmful impacts on the basic quality of daily life [6, 7]. Currently, it is estimated that more than 500 million people are afflicted with OA worldwide, causing an enormous health and economic burden globally [8]. To date, clinic treatments for OA patients include non-pharmacological approaches, pharmacological intervention, and eventual total joint replacement [9, 10]. Pharmacologic treatments have been investigated extensively and some agents have shown promising results in previous works. However, none of them has emerged with any substantial success and no disease-modifying OA drugs (DMOADs) were available to alleviate cartilage degeneration in the clinic currently. Therefore, there is a great unmet medical demand for exploring effective treatments for alleviating OA progression.

Cartilage progenitor cells (CPCs) are recognized as a novel cell population which harvested from the superficial layer of articular cartilage [11]. Some studies demonstrated that CPCs possess multi-directional differentiation ability, especially the chondrogenic potential [12–14]. Previous research has confirmed that in vitro cultured CPCs are proliferative and clonogenic, and present high expressions of stem cell biomarkers [13, 15, 16]. Convincing evidences have shown that CPCs play essential part in development and maturation of articular cartilage, and response to cartilage injury and OA progression [17, 18]. A recent study revealed that CPCs possess stronger migration ability, proliferative capacity, and chondrogenic potential than that of tissues-derived mesenchymal stem cells (MSCs) in vitro. Additionally, in vivo studies further confirmed that CPCs combined with platelet-rich plasma (PRP) scaffold showed superiority over bone marrow derived-MSCs combined with PRP scaffold in cartilage regeneration by using a rabbit articular cartilage defect model (critical size, 5 mm) [19]. Moreover, another study has supported the idea that CPCs have dominance in cell proliferative ability and chondrogenic differentiation capacity as seeding cells on cartilage repair and regeneration, compared with mature chondrocytes and bone marrow derived-MSCs [20]. Previous comparative research also demonstrated that CPCs are superior to MSCs for application in cartilage regeneration because of their lower expression of collagen X (a biomarker of hypertrophy cartilage) [19–21]. In general, CPCs have been considered as promising cell source for cartilage regeneration and OA treatment owing to their chondrogenic ability and inherent properties. However, there are still many disadvantages in stem cell transplantation that should be overcome, such as the risk of immune rejection and potential tumorigenesis [22]. Therefore, developing a superior strategy that can make full use of the benefits of CPCs without the potential risk of direct usage is highly essential.

There is growing evidence that extracellular vesicles (EVs) are primary bioactive factors of stem cells-based therapy [23]. EVs are natural nanosized particles (30–150 nm) that mediate cellular communication and regulate the behavior of recipient cells through delivering functional proteins, RNAs, and lipids [23, 24]. Recently, stem cells-derived EVs are considered as a promising therapeutic strategy in regenerative medicine, for their prominent pro-regenerative effects and low risk of immune rejection [25, 26]. Nevertheless, to date, the therapeutic function of CPCs-derived EVs (CPCs-EVs) for cartilage repair and OA treatment is barely reported. A recent study demonstrated that mouse-sourced CPCs-EVs promoted chondrocyte migration and proliferation in vitro. Moreover, intra-articular injection of CPCs-EVs attenuated cartilage degeneration in surgically-induced OA mice by delivering miRNA 221-3p [27]. Therefore, CPCs-EVs could be used as alternative for cartilage regeneration and OA treatment. We speculate that CPCs-EVs might have chondro-protective effects like their parental CPCs. Since CPCs-EVs can be obtained from in vitro cultured CPCs which is convenient for industrial production, and it holds great potential in OA treatment. However, the specific molecular mechanism involving in maintaining matrix homeostasis and inhibiting inflammatory response in OA cartilage remain elusive.

STAT3 is a well-known bioactive factor which involved in cell survival, proliferation, inflammatory response, and many other biological effects [28]. The pathogenic role of STAT3 signaling pathway has been widely investigated in different inflammatory diseases including OA [28, 29]. Previous studies have confirmed that activation of STAT3 decreased the expression of cartilage anabolism marker collagen II and caused cartilage matrix loss [29]. However, inhibition of STAT3 activation reversed the up-regulation of a disintegrin and metalloprotease with thrombospondin motifs (ADAMTs) and matrix metalloproteinases (MMPs), and further maintained the matrix homeostasis in articular cartilage [30, 31]. The results indicated that the STAT3 signaling pathway is involved in OA progression. For another, numerous studies have identified that STAT3 is a critical pro-inflammatory factor in OA progression [28, 29]. Under the stimulation of a classical in vitro OA inducer IL-1β on chondrocyte, STAT3 was quickly phosphorylated and the expressions of inflammatory cytokines (TNFα and IL6) were increased. Inhibition the phosphorylation of STAT3 effectively attenuated inflammatory response and decreased the expressions of inflammatory cytokines in OA cartilage [32–34]. These above studies suggested that STAT3 is an important signaling pathway that mediates matrix homeostasis and inflammatory response in chondrocytes, and STAT3 is an essential target for OA therapy. However, whether STAT3 signaling pathway plays a role in the regulative effects of CPCs-EVs on OA cartilage need further investigation.

The primary aim of the present study was to systematically evaluate the chondro-protective effects of CPCs-EVs in vitro and in vivo. First, we explored the protective effects of CPCs-EVs on chondrocytes in vitro. We found that CPCs-EVs exhibited protective effects in IL-1β-induced chondrocytes. Moreover, our results demonstrated that STAT3 signaling inhibition was correlated with the chondro-protective effects of CPCs-EVs. Liquid chromatography-tandem mass spectrometry analysis identified 991 proteins encapsulated in CPCs-EVs, including 77 new-found proteins beyond current Vesiclepedia database. By bioinformatics analysis, we showed that STAT3 regulatory proteins were enriched in CPCs-EVs and could be transported to chondrocytes. To enhance the protective effects of CPCs-EVs in vivo, CPCs-EVs were then incubated with cationic peptide ε-polylysine-polyethylene-distearyl phosphatidylethanolamine (PPD) for reversing surface negative charge. In anterior cruciate ligament transection (ACLT)-induced posttraumatic OA mice, our results indicated PPD modified CPCs-EVs (PPD-EVs) enhanced cartilage extracellular matrix production, inhibited inflammatory response, reduced OA-related pain and articular cartilage degeneration in mice. In ex-vivo cultured OA cartilage explants, PPD-EVs effectively promoted matrix production and inhibited inflammation. In conclusion, our findings underscore the potential of CPCs-EVs-based therapy as a promising strategy for OA treatment.

## Materials and methods

### Materials

Dulbecco’s modified Eagle’s medium (DMEM)-F12 and phosphate buffered saline (PBS) were obtained from HyClone (USA). Collagenase type II, penicillin-streptomycin (P.S.), amphotericin-B, and fetal bovine serum (FBS) were purchased from Gibco (USA). Bovine Serum Albumin (BSA), fibronectin, dimethyl sulfoxide (DMSO), ascorbic acid, and L-glutamine were supplied by Sigma (USA). 0.25% trypsin was obtained from Invitrogen (USA). Fibroblast growth factor-2 (FGF-2) and transforming growth factor-beta 2 (TGF-β2) were obtained from Biovision (USA). Interleukin-1β (IL-1β) was purchased from the R&D system (USA). εPL was purchased from Bainafo Bioengineering (China) and succinate ester of DSPE-PEG (DSPE-PEG-NHS) was synthesized by SinoPEG Bioengineering (China). STAT3 activator Colivelin TFA (Coli) and amphotericin-B were purchased from MedChemExpress (MCE, China). 4% paraformaldehyde (PFA) was obtained from servicebio (China). 4′,6-diamidino-2-phenylindole (DAPI) was supplied by the Beyotime Biotechnology (China). Cell Counting Kit-8 (CCK-8) was purchased from the Dojindo Molecular Technology (Japan).

### Isolation and identification of CPCs-EVs

#### Ethical approval

for this work was approved by the Animal Research Committee of Shanghai Sixth People’s Hospital Affiliated to Shanghai Jiao Tong University School of Medicine (Approval Number: SYXK2021-0028, Shanghai, China). CPCs were isolated from the superficial layer of articular cartilage by fibronectin adhesion method as previously reported [16, 35, 36]. Briefly, mice articular cartilage from femoral head was digested with 0.25% trypsin for 30 min and 0.25% collagenase type II for 4 h to obtain single cells. Next, the harvested single cells were subjected to differential adhesion on 10 g/ml fibronectin pre-coated plate in DMEM-F12 culture medium at a concentration of 400 cells/ml for 30 min. Subsequently, the culture medium and non-adherent cells were removed and replaced with standard growth media (DMEM-F12 medium containing 10% FBS, 2.5 mM/l L-glutamine, 2 g/ml amphotericin-B, 62 g/ml ascorbic acid, and 100 IU/ml P.S.). For another, the adherent cells were maintained to obtain CPC colonies (> 32 cells) for 12 days at 37 °C. Then the CPC clones were isolated and further expanded to the second passage (P2) with DMEM-F12 medium containing 10% FBS supplemented with 1 ng/ml TGF-2 and 5 ng/ml FGF-2, and 100 IU/ml P.S. To identify CPCs, cultured CPCs were observed at different time points (day 1, 3, 7, and 12) under a light microscope (Leica, Germany). Then the immunotypic phenotypes of CPCs were detected and analyzed by using flow cytometry. In particular, the antibodies studied against surface antigen labeled with fluorescein isothiocyanate (FITC) and phycoerythrin (PE) were CD29, CD44, CD105, CD90, CD45, CD34 and CD146 (Table S1). Briefly, single-cell suspension of CPCs was collected and incubated with 1% BSA. CPCs were then stained with the antibodies. The unbounded antibodies were then washed twice with 1% BSA. These cells were further analyzed by a CytoFLEX flow cytometer (Beckman Coulter Life Science, USA). Besides, immunofluorescence staining was also performed to detect the expressions of positive markers in CPCs including CD105 and PRX1 (Table S1). Briefly, cultured CPCs were first fixed with 4% PFA for 25 min at room temperature, and then permeablized with 0.25% Triton-X 100 for 30 min. Next, CPCs were blocked with 5% BSA for 30 min at room temperature and then incubated with primary antibodies against CD105 (1:200; Abcam) or PRX1 (1:100; Abcam) at 4 ℃ overnight. Subsequently, the cells were incubated with different secondary antibodies labelled with Alexa 546 or Alexa 488 for 1 h at room temperature and followed by incubation with DAPI staining solution for 10 min. Fluorescence images in all groups were captured and analyzed by using a laser scanning confocal microscopy (LSCM, Leica SP5II, Leica microsystems, Germany).

Next, CPCs-EVs were isolated from the collected culture supernatant by the classical differential ultracentrifugation method. Briefly, CPCs were cultured with serum-free DMEM-F12 culture medium for 12 h before ultracentrifugation. Then the supernatant was centrifuged at 300 × g for 10 min, 2,000 × g for 15 min, and 10,000 × g for 1 h to remove cells, dead cells, cell debris, and microvesicles. The supernatant was then ultracentrifuged at 100,000 × g for 114 min twice. After removing the supernatant, CPCs-EVs were resuspended in sterile PBS. Then CPCs-EVs were characterized by a series of experiments [37]. First, a nano-flow cytometry (N30 NanoFCM, China) was used to detect the size distribution and particle concentration of CPCs-EVs. Next, transmission electron microscope (TEM) analysis was used for morphology observation of CPCs-EVs. Briefly, CPCs-EVs suspensions were dropped onto a formvar-carbon-coated grid and dried in air for 20 min. Then, the grids were rinsed with sterile PBS and fixed in 1% (w/v) glutaraldehyde for 5 min. The grids were further rinsed with deionized water and stained with 2% (w/v) uranyl oxalate for 5 min. After drying, the microstructure of CPCs-EVs were observed by TEM (HitachiH-7650, Japan). The surface charge of CPCs-EVs was detected by a nanoparticle analyzer (DelsaMax Pro, Beckman Coulter, USA). Protein concentration of CPCs-EVs was quantified by the BCA Protein Assay Kit (Thermo Fisher Scientific, USA). Briefly, CPCs-EVs were digested with RIPA lysis buffer (Beyotime, China) and the protein was harvested following the manufacturer’s instruction. Next, a total of 10 µl protein sample was loaded into each well of a 96-well-plate and 200 µl of the working reagent was added. Then the plate was incubated for 30 min at 37℃ and the absorbance was measured at 562 nm by a multifunctional microplate reader (Varioskan LUX, Thermo Fisher Scientific, USA). To detect the protein expressions of surface markers in CPCs-EVs, the protein samples were collected from CPCs-EVs and CPCs using RIPA lysis buffer (Beyotime, China). The western blot analysis was performed as described previously [37]. Briefly, after being incubated with 5% BSA at room temperature for 60 min, the polyvinylidene difluoride (PVDF) membranes were incubated with primary antibodies including CD9 (1:1000), CD63 (1:1000), and TSG101 (1:1000) (Table S1) at 4℃ overnight. Then the membranes were incubated with horseradish peroxidase (HRP)-labeled secondary antibody at room temperature for 60 min and chemiluminescent signals were visualized by the ECL Western Blot detection kit and Bio-RAD imaging system (Bio-RAD, USA).

### Chondrocyte culture and treatment

Chondrocytes were obtained from articular cartilage. Briefly, as previously described [38, 39], the harvested articular cartilage was washed with PBS and cut into 1 mm^3^ by sterile scalpel, followed by 30 min incubation with 0.25% trypsin and 4 h digestion with 0.25% collagenase type II at 37 ℃. The dissociated chondrocytes were seeded at a density of 2 × 10^5^ cells/ml in DMEM-F12 complete culture medium supplemented with 10% FBS and 1% P.S. Chondrocytes at P2 were used in the following study. Subsequently, 10 ng/ml IL-1β was applied to chondrocytes for inducing OA inflammatory responses in vitro as previous reported [37, 40], and then treated with CPCs-EVs for 48 h.

### Measurement of sulfated glycosaminoglycan (GAG) and collagen content

The detection of collagen and GAG content in each group were applied as previously described [20, 41]. After CPCs-EVs treatment in cultured mouse cartilage explants for 48 h, the culture medium was removed, washed with PBS twice, and replaced with complete culture medium for 3 days. Then the DNA content in each group was detected against standard curves of calf thymus DNA (Sigma, USA). The collagen content in each group was analyzed by detecting hydroxyproline (HYP) content. Culture medium was allowed to evaporate to dryness. Residues were redissolved in sodium hydroxide solution, the vials sealed and incubated at 120 °C, opened and the contents again evaporated overnight. Residues were redissolved in distilled water. Then the sample was added to a well of a 96-well plate, chloramines-T solution was added and incubated for 5 min at room temperature. Next, p-dimethylaminobenzaldehyde/perchloric acid solution was added and incubated for 35 min at 65 °C. Plate was allowed to cool and read at 560 nm and HYP content calculated using a standard curve constructed from L-HYP (Sigma, USA) dissolved in distilled water in the range from 0 to 160 µg/ml. By using the dimethylmethylene blue (DMMB) assay (Sigma, USA), the total sulfated GAG content in each group was detected. Subsequently, the GAG content was detected and analyzed according to the standard curve which acquired from the chondroitin 6-sulfate (Sigma, USA). The content of GAG and collagen were both normalized by the content of DNA.

### Immunofluorescence staining

For immunofluorescence staining, cultured cells were first fixed with 4% PFA for 25 min at room temperature, and then permeablized with 0.25% Triton-X 100 for 30 min. Next, the cells were blocked with 5% BSA for 30 min at room temperature, and then incubated with primary antibodies against Ki-67 (1:200; Abcam), Collagen II (1:200; Abcam), and MMP13 (1:200; Abcam) at 4 ℃ overnight (Table S1). Subsequently, the cells were incubated with different secondary antibodies labelled with Alexa 546 or Alexa 488 for 1 h at room temperature and followed by incubation with DAPI staining solution for 10 min. Fluorescence images in all groups were captured and analyzed by using a laser scanning confocal microscopy (LSCM, Leica SP5II, Leica microsystems, Germany).

### RNA extraction and real-time quantitative polymerase chain reaction (RT-qPCR) analysis

Total RNA was extracted from chondrocytes in each group using Trizol (Invitrogen). The total RNA concentration in each group was measured by using a NanoDrop spectrophotometer (Thermo Fisher Scientific). Reverse transcription and RT-qPCR analysis were performed in triplicate as previously described [42, 43]. Reverse transcription was performed with reverse transcriptase (Takara, Japan) according to the manufacturer’s instruction and quantitative real-time PCR was performed using SYBR-green reagent (Yeasen Biotechnology, China) with the appropriate primers by the ABI Prism 7900HT Real Time System (Applied Biosystems, CA) according to the manufacturer’s instruction. By correcting the Ct (threshold cycles) values of the target genes to the Ct value of β-actin, ΔCt value in each group was calculated and the relative expressions of all target genes were expressed as 2^ΔΔCt^ with respect to the control group. Mouse and human primers used in this research were synthesized by Sangon (Shanghai, China) and listed in Table S2 and Table S3.

## CCK-8 assay

For cell proliferation assay, chondrocytes were seeded at a density of 6,000 cells/well on a 24-well plate. First, chondrocytes were treated with CPCs-EVs at different concentrations (1 × 10^7^ particles/ml, 1 × 10^8^ particles/ml, 1 × 10^9^ particles/ml, and 1 × 10^10^ particles/ml). Then, IL-1β-induced cells were treated with CPCs-EVs (1 × 10^9^ particles/ml) for different time points (24, 48, and 96 h). We added 40 µl CCK-8 solution to each well and the cells were incubated for 2–4 h in the dark. The absorbance of each well was detected by a multi-functional microplate reader (Thermo Fisher Scientific, USA) at 450 nm. The cell viability in each group was calculated using the equation: Cell viability (%) = (Absorbance sample/Absorbance control) × 100.

## ELISA analysis

To evaluate the levels of IL6, TNFα, PGE2 in the supernatants from chondrocytes, ELISA analysis was performed. Briefly, following the treatment of CPCs-EVs, the culture medium was collected for detecting the concentrations of IL6, TNFα, PGE2 using an ELISA detection kit (Shanghai Westang Bio-Tech, China) according to the manufacturer’s instruction. A microplate reader (Thermo Fisher Scientific, USA) was applied to measure the absorbance in each group at 450 nm.

### Western blot analysis

Chondrocytes were lysed in RIPA lysis buffer containing protease inhibitor and/or a phosphatase inhibitor cocktail. The total cell lysates were prepared in lysis buffer (150 mM NaCl, 1% Nonidet P-40, 50 mM Tris, 5 mM NaF), separated by SDS polyacrylamide gel electrophoresis, and transferred to the PVDF membrane. After blocking with 5% BSA in 0.1% Tween 20 TBS (TBST), the membranes were incubated with the primary antibodies overnight at 4 °C. After washing three times with TBST, the PVDF membranes were incubated with secondary antibody for 1 h and visualized by using the Bio-RAD imaging system (Bio-RAD, USA). The following primary antibodies were used: STAT3 (1:1000; Abcam), p-STAT3 (1:1000; Abcam), IL6 (1:1000; Abcam), TNFα (1:1000; Abcam), and β-actin (1:1000; Abcam). Related information about these antibodies were listed in Table S1.

### PPD-EVs construction and characterization

To achieve surface charge reverse, isolated CPCs-EVs were incubated with the PBS solution containing 100 µg/ml PPD for 60 min at room temperature as described previously [37]. After incubation, 1 ml mixed solution were diluted with 100 ml sterile PBS to eliminate PPD aggregation. Then, the diluted solutions were purified by ultracentrifugation to obtain PPD-EVs. To validate whether PPD modification could alter the integrity of CPCs-EVs, a series of experiments were performed. First, TEM was also used for morphology observation of PPD-EVs. Surface charge of PPD-EVs was detected by the nanoparticle analyzer. Then PPD-EVs suspension was added to the nano-flow cytometry, and the size distribution were evaluated by this machine.

### Cartilage penetration ability and joint retention time evaluation of PPD-EVs

Articular cartilage explants were harvested from the knee joints of OA patients by using an electric drill. After extraction, the cartilage explants were washed with PBS containing 1% P.S. In order to evaluate the cartilage uptake and penetration capacity of CPCs-EVs or PPD-EVs, lipophilic dye DiO was applied to label CPCs-EVs and PPD-EVs. Then a one-way transport teflon mould was used to evaluate the cartilage penetration ability of CPCs-EVs and PPD-EVs as described previously [37]. Briefly, the cartilage explants were stuck into the hole in the removable baffle which divided the mould to two chambers. 200 µl DiO labelled CPCs-EVs (1 × 10^9^ particles/ml) or PPD-EVs (1 × 10^9^ particles/ml) were added to one side chamber of the mould, while 200 µl sterilized PBS was added to the other chamber of the mould. After penetration for 2 days, the cartilage explants were immersed with OCT glue and sectioned to 5 μm slides by a vibrating microtome (Leica, Germany) followed by an immediate microscopy observation (Leica Microsystems, Germany).

For joint retention time evaluation of CPCs-EVs and PPD-EVs [37], a near-infrared fluorescence lipid dye DiR was used to label CPCs-EVs and PPD-EVs, and the IVIS Spectrum imaging system (PerkinElmer, USA) was applied for analysis. Briefly, 10 µl DiR-labelled CPCs-EVs (1 × 10^9^ particles/ml) or PPD-EVs (1 × 10^9^ particles/ml) was injected to the mouse knee joint cavity. After injection, images of each mouse joint were recorded by the IVIS Spectrum imaging system for a 14-day period.

#### Ethical approval and experimental animals

Animal experiments are reported following the ARRIVE guidelines. All animal work in this research was approved by the Animal Research Committee of Shanghai Sixth People’s Hospital Affiliated to Shanghai Jiao Tong University School of Medicine (Approval Number: SYXK2021-0028, Shanghai, China). All animal studies were performed in the animal room of Shanghai Sixth People’s Hospital Affiliated to Shanghai Jiao Tong University School of Medicine. To establish ACLT surgery-induced animal post-traumatic OA model, 8-week-old C57BL/6 male mice (20–22 g) were used in our study and were housed five per cage in standard specific pathogen-free condition. A total of 20 mice were randomly classified into 4 groups: ACLT + PBS group (*n* = 5; treated with 10 µl PBS), ACLT + CPCs-EVs (10^9^) group (*n* = 5; treated with 10 µl 1 × 10^9^ particles/ml CPCs-EVs), ACLT + PPD-EVs (10^9^) group (*n* = 5; treated with 10 µl 1 × 10^9^ particles/ml PPD-EVs), and Sham group (*n* = 5; treated with 10 µl PBS). Mice were subjected to sham surgery or ACLT surgery as described previously [37]. Briefly, anesthesia was induced by 2% isoflurane in high-flow oxygen, then the right joint capsule was opened and the anterior cruciate ligament was transected to destabilize the right knee joint in ACLT group mice, whereas the joint capsule was opened without any other operation in Sham group mice. All surgery was performed by the same surgeon (K.F.). For treatments, 10 µl PBS, CPCs-EVs (1 × 10^7^ particles), or PPD-EVs (1 × 10^7^ particles) was injected into the knee joint of ACLT-induced OA mice once a week after 4 weeks of surgery. Mice in Sham group were treated with 10 µl PBS as a control. After treatments for 6 weeks, all mice were anesthetized and euthanized with sodium pentobarbital (50 mg/kg, intraperitoneally).

### Histological and immunohistochemical (IHC) analysis

The mice knee joints in each group were harvested and fixed in 4% PFA solution at room temperature overnight, decalcified with 10% EDTA solution for 7 d, embedded in paraffin, and sectioned at 5 μm thickness. Then the samples were stained to evaluate the cartilage destruction and proteoglycan deposition in each group by using the Alcian Blue Cartilage Staining Kit (Solarbio, China), the Modified Safranin O/fast green Cartilage Staining Kit (Solarbio, China), and Hematoxylin-Eosin (H&E) Staining Kit (Solarbio, China). The Osteoarthritis Research Society International (OARSI) score of cartilage destruction in each group was analyzed as previously reported [44]. The medial tibial bone sclerosis (grade − 5 ∼ 5) in each group was scored by assessing the subchondral trabecular bone to marrow ratio [45]. Synovial inflammation in each group was scored on an arbitrary scale from 0 to 3 depending on infiltration of inflammatory cells into the synovial membrane by H&E Staining [46].

For IHC staining, cartilage sections in all groups were firstly deparaffinized in xylene, hydrated through ethanol series, and repaired by using pepsin antigen repair solution. Then, each cartilage section was blocked with 5% BSA for 1 h, and incubated overnight at 4 ℃ with different primary antibodies including Collagen II (1:100; Abcam), PRG4 (1:100; Abcam), MMP13 (1:200; Abcam), ADAMTS5 (1:200; Abcam), IL-1β (1:100; Abcam), IL6 (1:100; Abcam), TNFα (1:100; Abcam), and p-STAT3 (1:500; Abcam) (Table S1). Subsequently, HRP-labelled secondary antibodies and antigen-positive chondrocytes were visualized by using the DAB Substrate kit (Servicebio, China).

### OA pain analysis

According to previously reported method [37, 47], hind-paw withdrawal threshold of each mouse was examined to evaluate OA-related pain. In particular, the mouse was placed in an elevated metal grid cage individually with enough space to move their paws freely, and the rest body was strictly restricted with the plastic plate. Subsequently, the right hind-paw withdrawal threshold of each mouse was detected by using an electronic von Frey instrument (BIO-EVF4, France). In brief, the probe tip was placed perpendicularly into the mid-plantar surface of the mouse paw gently and the pressure (0–100 g) was increased steadily until the mouse hind paw was lifted. The hind-paw withdrawal threshold (g) was taken as the necessary pressure to first lift the paw and the lower withdrawal threshold value (g) of each mouse was recognized as the indicator of OA-related pain.

### Human articular cartilage harvest, culture, and treatment

All procedures in this research were approved by the Independent Ethics Committee of Shanghai Jiao Tong University Affiliated Sixth People’s Hospital (Approval Number: 2023-YS-012, Shanghai, China). Written informed consents were provided from all patients. Articular cartilage samples including the condyles were collected from seven female OA patients (mean ± S.D. age, 72.43 ± 5.12 years old; range, 65 to 81 years old) undergoing total knee joint replacement. OA was macroscopically diagnosed according to the Modified Outerbridge Classification [48]. Briefly, stage 0, normal articular cartilage; stage 1, softening of the articular cartilage; stage 2, fibrillation or superficial fissures of the cartilage; stage 3, deep fissuring of the cartilage without exposed subchondral bone; stage 4, exposed subchondral bone. Specimens from severe damaged region (Stage 2 and 3) in osteoarthritic cartilage were defined as OA affected cartilage. Specimens from relatively heathy region (Stage 0) in osteoarthritic cartilage were defined as non-OA damaged cartilage.

For cartilage explants culture, explants were harvested from knee joint with OA cartilage morphology using biopsy punch (5 mm in diameter and 2 mm in thickness) and washed twice with PBS. Then cartilage explants were cultured in DMEM-F12 medium supplemented with 10% FBS and 1% P.S. in a 48-well-plate. Then, the OA cartilage explants were treated with PPD-EVs for 7 d.

### Proteomic analysis

The proteomic analysis of CPCs-EVs was performed by the Shanghai Oebiotech Company (Shanghai, China). First, 400 µl CPCs-EVs were digested in SDT lysis buffer (4% SDS, 100 mM Tris- HCl, 1 mM DTT, pH 7.6). The lysate was sonicated for 15 min and heated to 95℃ for 15 min. After centrifuged at 14, 000 × g for 40 min, the supernatant was quantified with the BCA Protein Assay Kit. 20 µg protein for each sample were mixed with 5 × loading buffer and boiled for 5 min. The protein was separated on 12.5% SDS-PAGE gel.

All analysis were performed by a Q-Exactive HF mass spectrometer (Thermo, USA) equipped with a Nanospray Flex source (Thermo, USA). Samples were loaded and separated by a C18 column (25 cm × 75 μm) on an EASY-nLCTM 1200 system (Thermo, USA). The flow rate was 300nL/min and linear gradient was 90 min (0 ∼ 1 min, 0-2% B; 1 ∼ 2 min, 2-6% B; 2 ∼ 51 min, 6-21% B; 51 ∼ 70 min, 21-31% B; 70 ∼ 81 min, 31-43% B; 81 ∼ 84 min, 43-100% B; 84 ∼ 90 min, 100%B; mobile phase A = 0.1% FA in water and B = 0.1% FA in 80% ACN and 19.9% water).

Full MS scans were acquired in the mass range of 350–1650 m/z with a mass resolution of 60,000 and the AGC target value was set at 3e6. The 20 most intense peaks in MS were fragmented with higher-energy collisional dissociation (HCD) with collision energy of 28. MS/MS spectra were obtained with a resolution of 30,000 with an AGC target of 2e5 and a max injection time of 80 ms. The Q Exactive HF dynamic exclusion was set for 40 s and run under positive mode.

The LC-MS/MS raw data were imported in Maxquant (Version1.6.17.0) for labeling free quantification analysis and the search engine was Andromeda. For limiting a certain number of peak matches by chance a target-decoy-based false discovery rate (FDR) approach is utilized. For peptide identification, mass and intensity of the peptide peaks in a mass spectrometry (MS) spectra are detected and assembled into 3D peak hills over the m/z retention time plane, which are filtered by applying graph theory algorithms to identify isotope patterns. High mass accuracy is achieved by weighted averaging and through mass recalibration by subtracting the determined systematic mass error from the measured mass of each MS isotope pattern. Peptide and fragment masses (in case of an MS/MS spectra) are searched in a specific sequence database, and are then scored by a probability-based approach termed peptide score. The assembly of peptide hits into protein hits to identify proteins is the next step. The assembly of peptide hits into protein hits to identify proteins is the next step, in which each identified peptide of a protein contributes to the overall identification accuracy. The organism specific database search includes not only the target sequences, but also their reverse counterparts and contaminants, which helps to determine a statistical cutoff for acceptable spectral matches.

Next, the identified CPCs-EVs proteins were compared with the results from the Vesiclepedia database by using the FunRich Software. Then the gene ontology (GO) enrichment analysis was processed by the FunRich Software and the canonical pathway analysis was also performed by using the Ingenuity Pathway Analysis.

### Statistical analysis

All statistical analysis were performed by GraphPad Prism version 8.0 for Windows (GraphPad Software, Boston, Massachusetts, USA, www.graphpad.com) and expressed as mean ± S.D. The two-tailed Student’s t-test was applied to assess the differences in means between two groups. One-way analysis of variance (ANOVA) was employed to detect differences among three or more groups. For the differences in means across three or more groups over time, two-way repeated measures ANOVA was used. The post hoc Bonferroni test was applied for pairwise comparisons between means when ANOVA showed significant differences. All experiments were repeated at least three times independently and representative experiment data are presented. *P* < 0.05 was considered statistically significant. Statistical significance was set as *P* < 0.001 (***, ^###^,^$$$^), *P* < 0.01 (**,^##^,^$$^), and *P* < 0.05 (*,^#^,^$^).

## Results

### Characterization of CPCs-EVs

First, the morphology of CPCs isolated from articular cartilage was detected by using a microscope. Colony formation was observed after 12 d culture of primary CPCs. CPCs were small, round or polygonal, and arranged in whorls or bundles (Fig. S1). In addition, CPCs expressed cell surface biomarkers that are found on the stem cells typically [49, 50]. The flow cytometry analysis demonstrated that CPCs were homogeneously positive for CD29 (99.70%), CD44 (99.70%), CD105 (95.50%) and CD90 (94.00%) (Fig. S2). CPCs were negative for CD45 (0.18%), CD34 (0.14%) and CD146 (0.40%) (Fig. S2). Immunofluorescence staining results showed the cells expressed CPCs specific markers, including CD105 and PRX1 (Fig. S3).

**Fig. 1:**
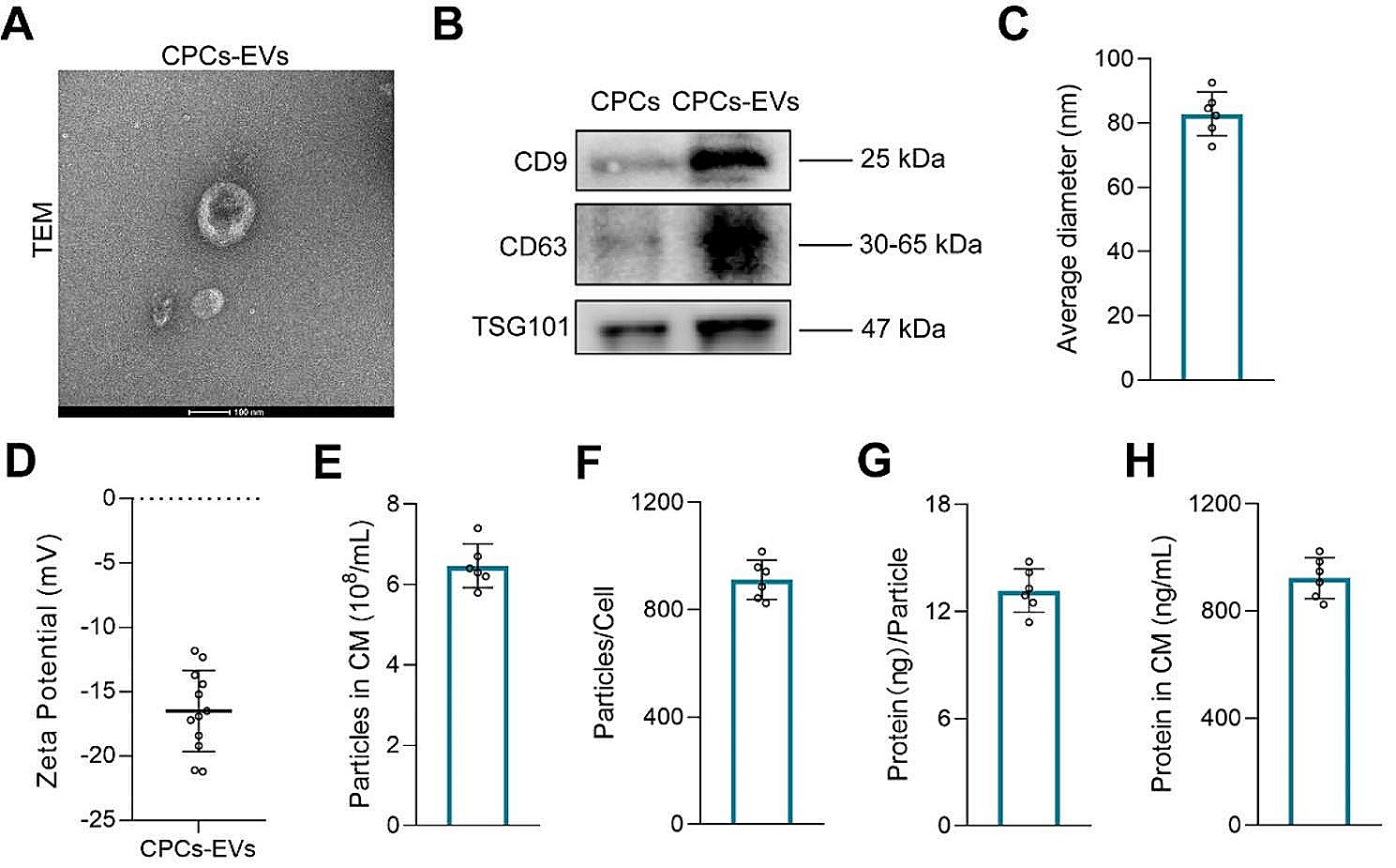
Characterization of CPCs-EVs. **A** Representative morphological image of CPCs-EVs captured by TEM. Scale bar = 100 nm. **B** The surface markers (CD9, CD63, and TSG101) of CPCs-EVs were detected by western blot analysis. **C** Average particle diameter of CPCs-EVs was detected by nano-flow cytometry. (*n* = 6). **D** The zeta potential of CPCs-EVs was detected by a nanoparticle analyzer. (*n* = 12). **E-H** Quantification of CPCs-EVs parameters, including the particle concentration and the protein concentration. (*n* = 6)

Next, we isolated the EVs from the culture supernatants of CPCs. TEM result indicated the purified CPCs-EVs displayed a size around 100 nm and a spherical shape (Fig. 1A). Moreover, we further detected the biomarkers of the CPCs-EVs by western blot analysis. As exhibited in Fig. 1B, CPCs-EVs expressed classical proteins that are commonly present in EVs, including CD9, CD63, and TSG101. The average particle size of CPCs-EVs was verified to be 82.87 ± 6.23 nm in diameter by nano-flow cytometry (Fig. 1C). The average zeta potential of CPCs-EVs was − 16.49 ± 3.01 mV (Fig. 1D). Additionally, the yield of CPCs-EVs was calculated in accordance with particle concentration and protein concentration. The mean particle concentration of CPCs-EVs was 6.47 × 10^8^ ± 0.49 × 10^8^ particles per ml in the culture medium (CM) (Fig. 1E) and 911.50 ± 67.42 particles per cell (Fig. 1F). Besides, the mean protein concentration of CPCs-EVs was 13.18 × 10^− 7^ ± 1.11 × 10^− 7^ ng per particle (Fig. 1G) and 923.33 ± 69.72 ng per ml in CM (Fig. 1H). Overall, these results demonstrated that the EVs were successfully purified and identified from CPCs.

### The chondro-protective effects of CPCs-EVs on IL-1β-induced chondrocytes

**Fig. 2:**
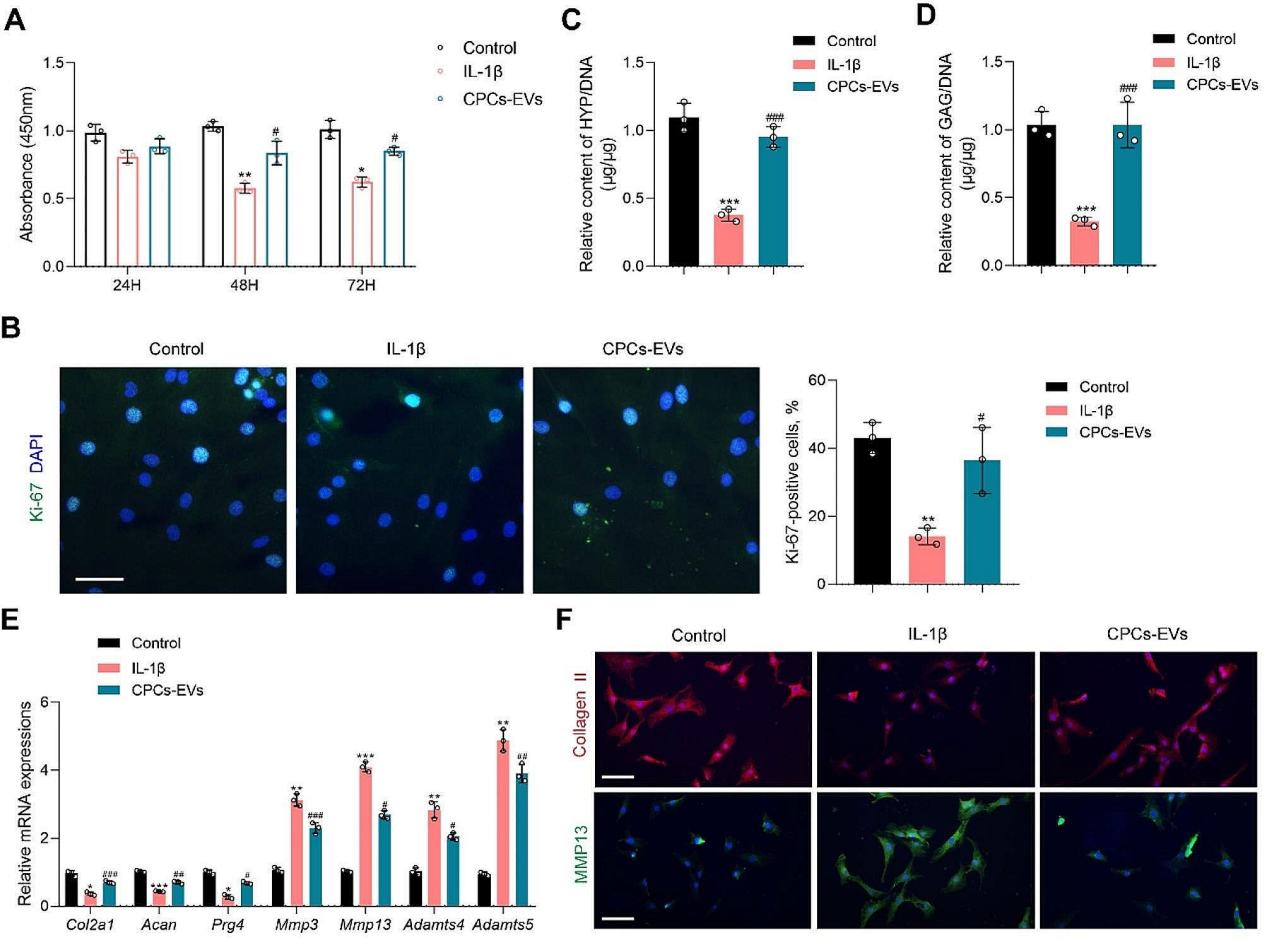
The protective effects of CPCs-EVs on IL-1β-induced chondrocytes. **A** Chondrocyte proliferative ability was detected by CCK-8 assay. (*n* = 3). **B** Immunofluorescence staining for Ki-67 (green) and percentage of Ki-67-positive cells in each group. (*n* = 3). Scale bar = 50 μm. **C** Relative HYP content was detected after CPCs-EVs treatment in IL-1β-induced chondrocytes. (*n* = 3). **D** Relative GAG content was detected after CPCs-EVs treatment in IL-1β-induced chondrocytes. (*n* = 3). **E** Relative mRNA expressions of anabolism-related genes (*Col2a1*, *Acan*, *Prg4*) and catabolism-related genes (*Mmp3*, *Mmp13*, *Adamts4*, *Adamts5*) were detected by PCR analysis. (*n* = 3). **F** Immunofluorescence staining for Collagen II (red) and MMP13 (green) in each group. (*n* = 3). Scale bar = 100 μm. Data represent mean ± S.D. *P < 0.05, **P < 0.01, ***P < 0.001 versus the Control group. ^#^*P* < 0.05, ^##^*P* < 0.01, ^###^*P* < 0.001 versus the IL-1β group

To evaluate the protective effects of CPCs-EVs on chondrocytes in vitro, we first compared the effects of CPCs-EVs at different concentrations on chondrocyte proliferation. Our results demonstrated that both 1 × 10^9^ particles/ml and 1 × 10^10^ particles/ml CPCs-EVs showed significant promoting effect on chondrocyte proliferation, and there is no difference between these two groups (Fig. S4). Therefore, CPCs-EVs at the concentration of 1 × 10^9^ particles/ml was used in our following studies. Next, we further evaluated the chondro-protective effects of 1 × 10^9^ particles/ml CPCs-EVs in IL-1β-induced chondrocytes. The results of CCK-8 assay demonstrated chondrocyte proliferative ability was inhibited after IL-1β treatment for 48 h, while CPCs-EVs effectively reversed this condition (Fig. 2A). Immunofluorescence staining for Ki-67 confirmed that 1 × 10^9^ particles/ml CPCs-EVs increased the ratio of Ki-67-positive proliferative cells in IL-1β-induced chondrocytes (from 14.10% ± 2.01–36.52% ± 7.94%), compared with the PBS-treated group (Fig. 2B). Together, CPCs-EVs exhibited proliferation-promoting ability in IL-1β-induced chondrocytes. Then, we evaluated the effects of 1 × 10^9^ particles/ml CPCs-EVs on cartilage matrix homeostasis in IL-1β-induced chondrocytes. First, the cartilage matrix GAG and collagen contents were detected in IL-1β-induced chondrocytes after CPCs-EVs treatment. Our results demonstrated that CPCs-EVs effectively promoted the deposition of collagen and GAG in IL-1β-induced chondrocytes (Fig. 2C, D). PCR analysis results also revealed that 1 × 10^9^ particles/ml CPCs-EVs effectively promoted the expressions of cartilage matrix anabolism genes (*Col2a1*, *Acan* and *Prg4*) and down-regulated the expressions of catabolism genes (*Mmp3*, *Mmp13*, *Adamts4* and *Adamts5*) (Fig. 2E). The result of immunofluorescence staining for Collagen II showed 1 × 10^9^ particles/ml CPCs-EVs effectively promoted the expression of cartilage matrix Collagen II in IL-1β-induced chondrocytes (Fig. 2F). Moreover, immunofluorescence staining for MMP13 further confirmed that CPCs-EVs inhibited cartilage matrix catabolism in IL-1β-induced chondrocytes (Fig. 2F). Altogether, our in vitro results demonstrated that 1 × 10^9^ particles/ml CPCs-EVs showed pro-anabolism effect and catabolism inhibitory effect in IL-1β-induced chondrocytes.

**CPCs-EVs alleviate inflammatory response by blocking STAT3 activation**in vitro.

**Fig. 3:**
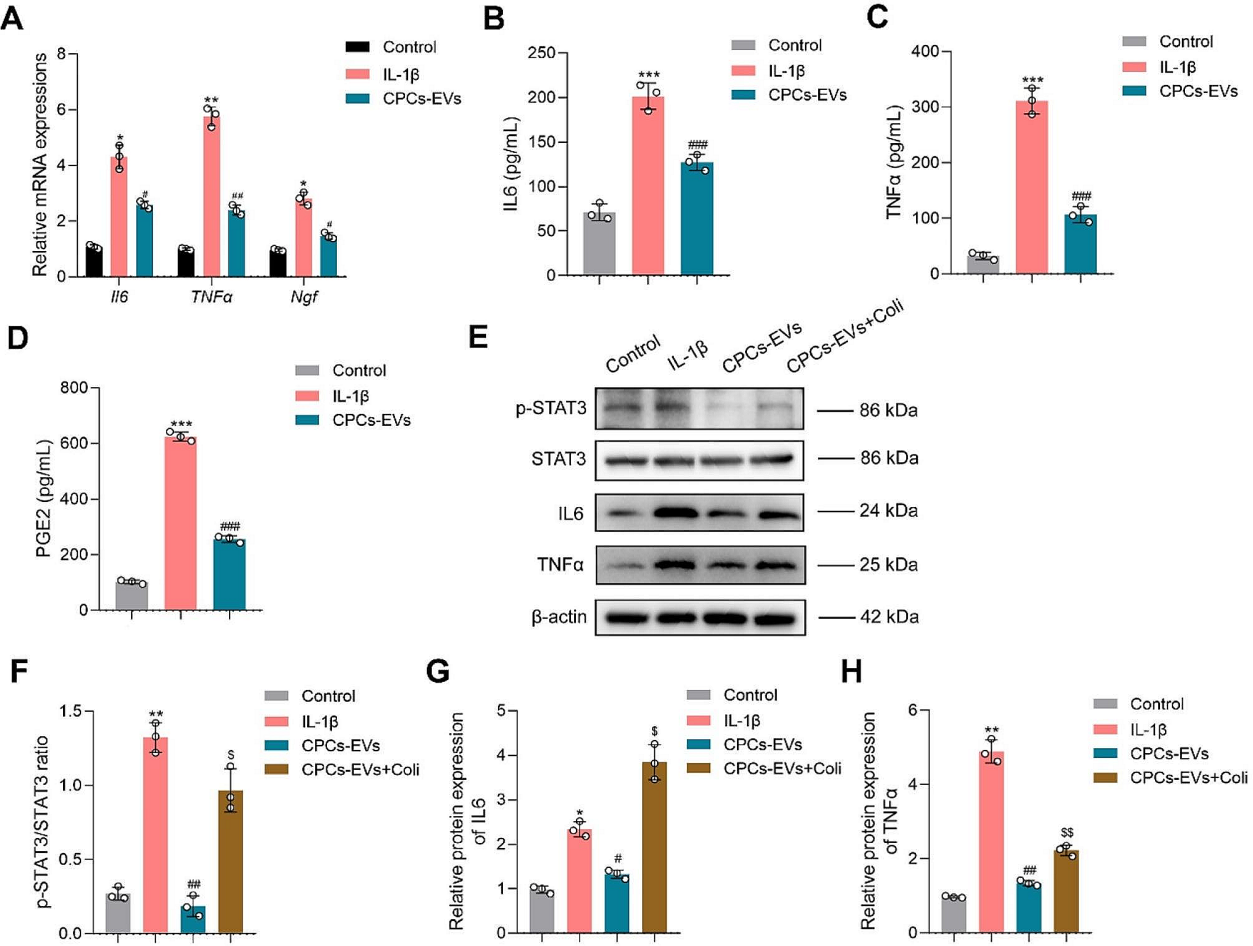
CPCs-EVs alleviate inflammatory response by inhibiting STAT3 activation in vitro. **A** Relative mRNA expressions of inflammatory cytokines (*Il6*, *TNFα*, *Ngf*) in mouse chondrocytes were detected by PCR analysis. (*n* = 3). **B** The concentration of IL6 was detected by ELISA assay in chondrocytes after different treatments. (*n* = 3). **C** The concentration of TNFα was detected by ELISA assay in chondrocytes after different treatments. (*n* = 3). **D** The concentration of PGE2 was detected by ELISA assay in chondrocytes after different treatments. (*n* = 3). **E** Western blot analysis of p-STAT3, STAT3, IL6, and TNFα expressions in chondrocytes after treatments. (*n* = 3). **F** Quantification of p-STAT3/STAT3 ratio in different groups. (*n* = 3). **G** Relative protein expression of IL6 in different groups. (*n* = 3). **H** Relative protein expression of TNFα in different groups. (*n* = 3). Data represent mean ± S.D. *P < 0.05, **P < 0.01, ***P < 0.001 versus the Control group. ^#^*P* < 0.05, ^##^*P* < 0.01, ^###^*P* < 0.001 versus the IL-1β group. ^$^*P* < 0.05, ^$$^*P* < 0.01 versus the CPCs-EVs group

Next, we further investigated whether the chondro-protective effects of CPCs-EVs were related to the regulation of STAT3 signaling pathway. First, our in vitro results demonstrated that CPCs-EVs decreased the mRNA expressions of inflammatory cytokines including *Il6*, *Tnfα*, and *Ngf* in chondrocytes (Fig. 3A). In addition, the concentrations of inflammatory cytokines were also detected by ELISA assay in vitro. Our results showed that CPCs-EVs significantly inhibited the secretion of inflammatory cytokines including IL6, TNFα, and PGE2 (Fig. 3B-D).

Subsequently, a classical STAT3 activator Colivelin TFA (Coli) was used to further confirm whether the chondro-protective effects of CPCs-EVs were associated with STAT3 inhibition. Our western blot analysis results demonstrated that CPCs-EVs decreased the protein expressions of IL6, TNFα, and p-STAT3 in IL-1β-induced chondrocytes (Fig. 3E-H), and down-regulated the ratio of p-STAT3/STAT3 (Fig. 3F). However, Coli treatment partially abrogated the inhibitory effect of CPCs-EVs on IL-1β-induced chondrocytes (Fig. 3E-H). Overall, these results suggested that STAT3 inhibition played an important role in the protective effects of CPCs-EVs on IL-1β-induced chondrocytes.

### Proteomics analysis of CPCs-EVs

**Fig. 4:**
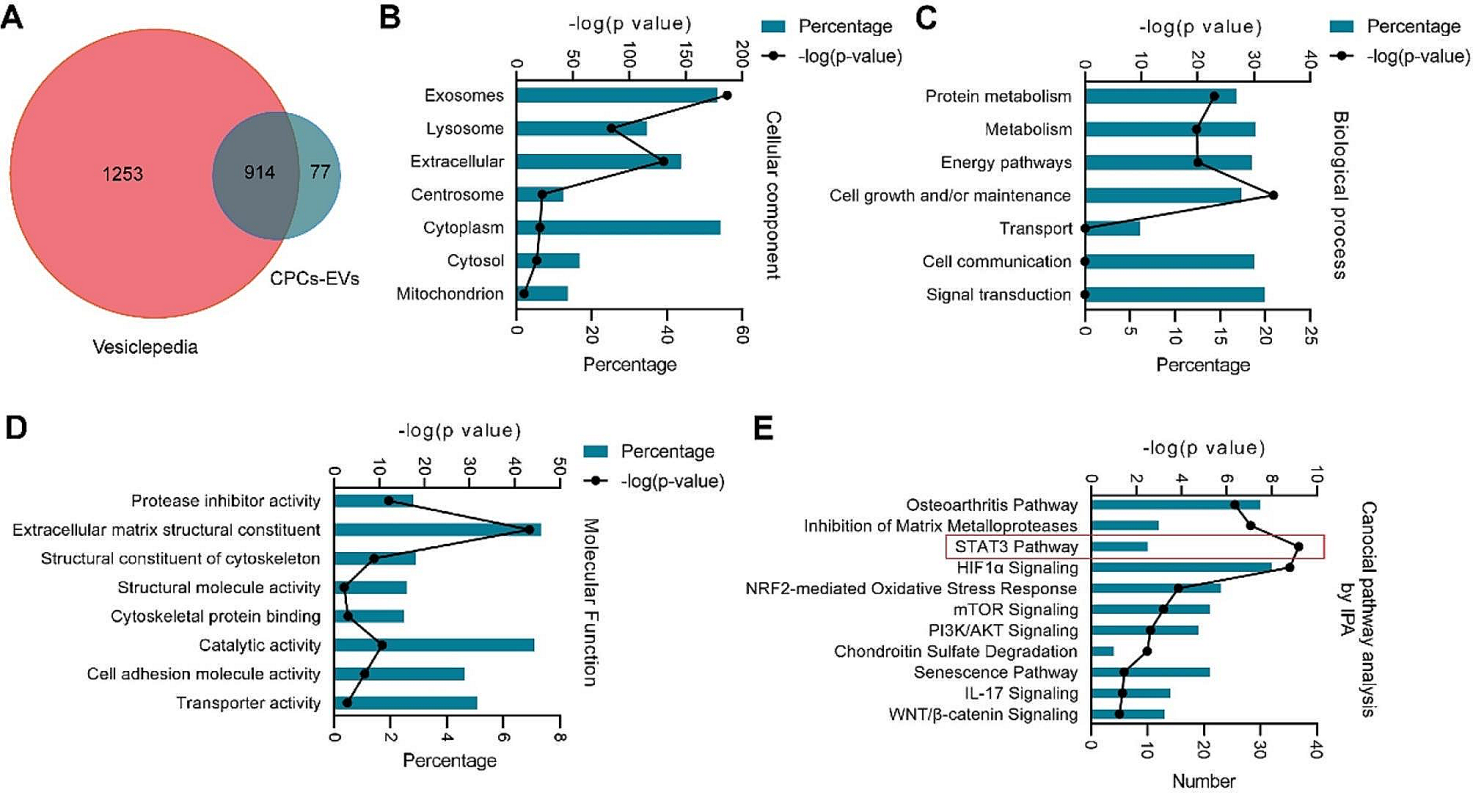
Proteomics analysis of CPCs-EVs. **A** Venn diagram showing the overlapping and unique proteins in CPCs-EVs with Vesiclepedia database. **B** Gene Ontology Cellular Component analysis of proteins enriched in CPCs-EVs. **C** Gene Ontology Biological Process analysis of proteins enriched in CPCs-EVs. **D** Gene Ontology Molecular Function analysis of proteins enriched in CPCs-EVs. **E** IPA displaying canonical pathway analysis of CPCs-EVs proteins

Previous studies have demonstrated that stem cells-derived EVs can regulate the biological processes of recipient cells through delivering encapsulated proteins. Herein, we further performed LC-MS/MS analysis and identified a total of 991 proteins in CPCs-EVs (Table S4). Among these identified proteins, 914 proteins can be matched to the Vesiclepedia database while 77 proteins were not included in the current database (Fig. 4A).

Next, Gene Ontology (GO) analysis of cellular component, molecular function, and biological process for CPCs-EVs were performed by using the FunRich Software. Cellular component analysis showed that 53.50% of the identified proteins were derived from exosomes and 54.28% of the identified proteins were derived from cytoplasm (Fig. 4B). Biological process analysis suggested an enrichment of proteins involved in “Signal transduction” (19.96%), “Metabolism” (18.88%), “Cell communication” (18.77%), “Energy pathways” (18.55%), and “Cell growth and/or maintenance” (17.37%) (Fig. 4C). Molecular function analysis revealed that “Extracellular matrix structural constituent” (7.33%) and “Catalytic activity” (7.12%) were two major components for the protein category of CPCs-EVs (Fig. 4D).

Subsequently, IPA analysis was performed to explore the potential canonical pathways in which identified CPCs-EVs proteins were involved. Our results showed that these proteins were associated with some signaling pathways including “Osteoarthritis Pathway”, “Inhibition of Matrix Metalloproteases”, “STAT3 pathway”, “HIF1α signaling”, “NRF2-mediated Oxidative Stress Response”, and others (Fig. 4E). Among these signaling pathways, “STAT3 signaling”, “HIF1α signaling”, “mTOR signaling”, “PI3K/AKT signaling”, and “WNT/β-catenin signaling” were related to the regulation of inflammatory response. According to our in vitro and in vivo results, we further confirmed that there are 10 proteins which can regulate STAT3 pathway were encapsulated in CPCs-EVs, including EGFR, FGFR1, FLT4, HGF, IGF1, IGF2R, RAP1B, TGFB1, TGFB2, and TGFBR3. These results indicated that CPCs-EVs could deliver the encapsulated regulative proteins to chondrocytes, modulating STAT3 pathway, thereby inhibiting inflammation and maintaining cartilage matrix homeostasis.

#### Positively charged CPCs-EVs maintain cartilage matrix homeostasis and alleviate OA progression in ACLT-induced mice

Next, to enhance the bioavailability and cartilage penetrating capacity of CPCs-EVs in vivo, CPCs-EVs were incubated with PPD for surface charge reverse. PPD modification successfully reversed the surface charge of CPCs-EVs, and it showed no obvious influence on the integrity of CPCs-EVs (Fig. S5). Given the abundant negatively charged biomacromolecules in cartilage matrix, human articular cartilage explants were used to evaluate whether PPD modification could enhance the penetration ability of CPCs-EVs. Our results showed CPCs-EVs still concentrated in the superficial zone of the cartilage explants after penetration for 2 days, suggesting a very limited penetration ability (Fig. S6A). In contrast, PPD-EVs had distributed throughout the cartilage explants after penetration for 2 days (Fig. S6A). Next, DiR labelled CPCs-EVs or PPD-EVs were injected into the mouse knee joint to assess the retention capacity. In vivo imaging analysis was used to further evaluate the joint retention time of CPCs-EVs and PPD-EVs. Notably, the fluorescence signal was still detectable in PPD-EVs group at day 14 after intra-articular injection, while the fluorescence signal was barely detectable in CPCs-EVs group at day14 after injection, indicating that the retention time of PPD-EVs in knee joint is much longer than that of CPCs-EVs (Fig. S6B). These results demonstrated that PPD modification significantly extended the joint retention ability and enhanced the cartilage penetration capacity of CPCs-EVs (Fig. S6).

**Fig. 5:**
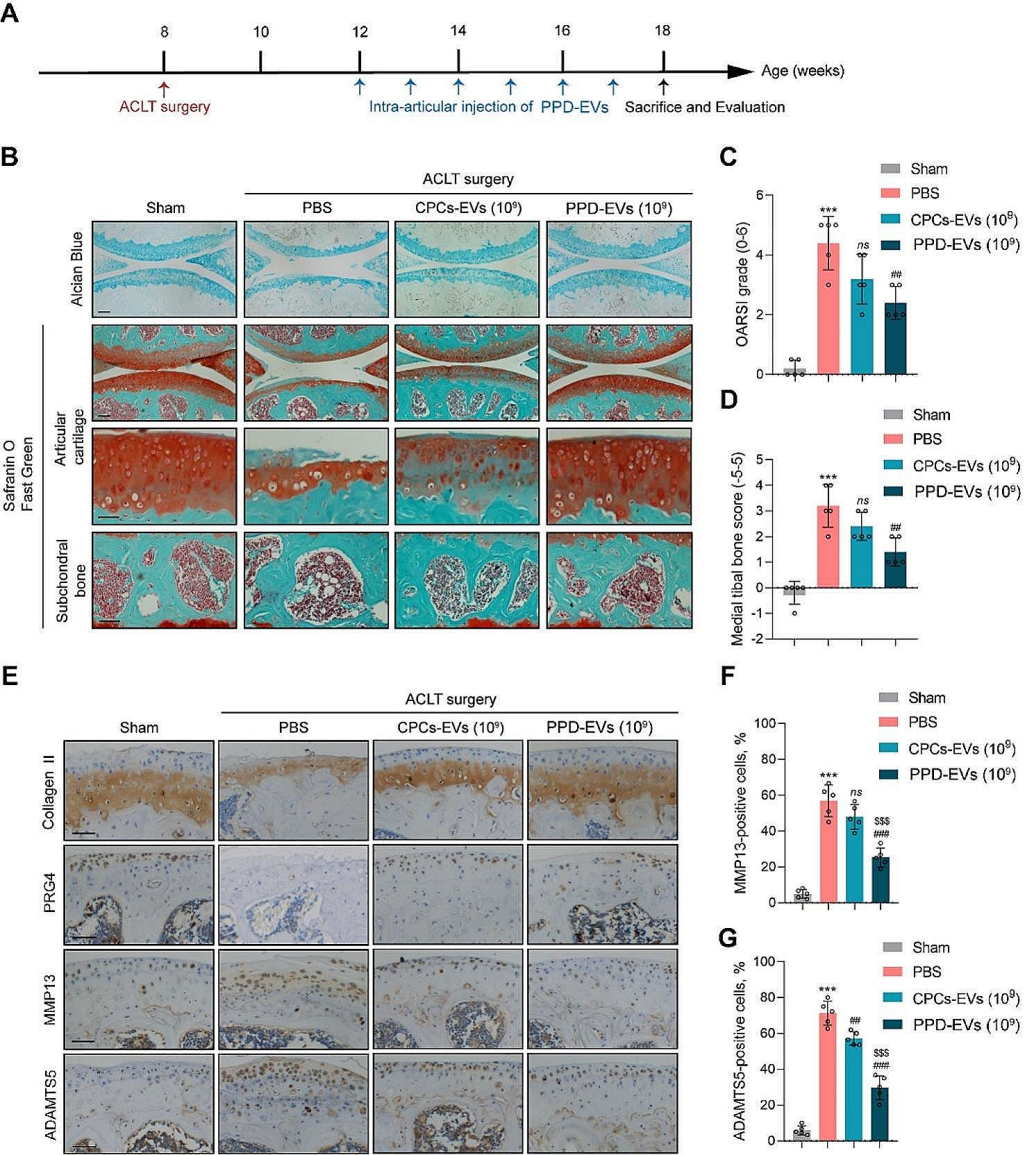
The therapeutic effects of PPD-EVs in ACLT-induced posttraumatic OA mice model. **A** Schematic of the time course for the experiments in ACLT-induced mice. Mice undergoing ACLT surgery were intra-articularly injected with different treatments and evaluated as indicated. **B** Light microscopy images of articular cartilage stained by the Alcian Blue and Safranin O-Fast Green in all groups were observed and analyzed. Scale bar = 100 μm and 50 μm. **C** Cartilage loss and destruction in each group was evaluated scored by the OARSI scoring system. **D** The score of medial tibial bone in each group was evaluated. **E** IHC staining for Collagen II, PRG4, MMP13, and ADAMTS5 in mice after treatments. Scale bar = 100 μm. **F** Quantification of MMP13-positive cells in articular cartilage after different treatments. **G** Quantification of ADAMTS5-positive cells in each group. Data represent mean ± S.D. ***P < 0.001 versus the Sham group. ^##^*P* < 0.01, ^###^*P* < 0.001 versus the HA group. ^$$$^*P* < 0.001 versus the CPCs-EVs (10^9^) group. *ns*, not significant

Then we further explored whether positively charged PPD-EVs could exert chondro-protective effects in ACLT-induced posttraumatic OA mice. Four weeks after ACLT surgery, CPCs-EVs or PPD-EVs at the concentration of 1 × 10^9^ particles/ml were injected into the knee joint of mice once a week (Fig. 5A). After treatment for six weeks, we found 1 × 10^9^ particles/ml CPCs-EVs and PPD-EVs both effectively promoted extracellular matrix anabolism, reduced cartilage destruction and subchondral bone sclerosis in ACLT-induced OA mice (Fig. 5B). Compared with the PBS-treated group, the OARSI score in ACLT mice was declined from 4.40 ± 0.80 to 2.4 ± 0.49 after 1 × 10^9^ particles/ml PPD-EVs treatment, while there is no difference between the PBS-treated group and 1 × 10^9^ particles/ml CPCs-EVs-treated group (Fig. 5C). Additionally, the medial tibial bone score in ACLT-induced mice was also effectively down-regulated from 3.20 ± 0.75 to 1.40 ± 0.49 after the intervention of 1 × 10^9^ particles/ml PPD-EVs, compared with the PBS-treated group (Fig. 5D). However, the medial tibial bone score in 1 × 10^9^ particles/ml CPCs-EVs-treated group showed no difference with the PBS-treated group (Fig. 3D).

IHC staining for anabolism-associated biomarkers (Collagen II and PRG4) and catabolism-related biomarkers (MMP13 and ADAMTS5) confirmed that CPCs-EVs effectively promoted cartilage matrix deposition and inhibited catabolism in ACLT-induced mice, and 1 × 10^9^ particles/ml PPD-EVs showed a stronger matrix homeostasis maintaining effect than 1 × 10^9^ particles/ml CPCs-EVs (Fig. 5E-H). Overall, our results verified that PPD-EVs successfully maintain cartilage matrix homeostasis in ACLT-induced OA mice.

### PPD-EVs attenuate joint inflammation and OA-related pain in ACLT-induced mice

**Fig. 6:**
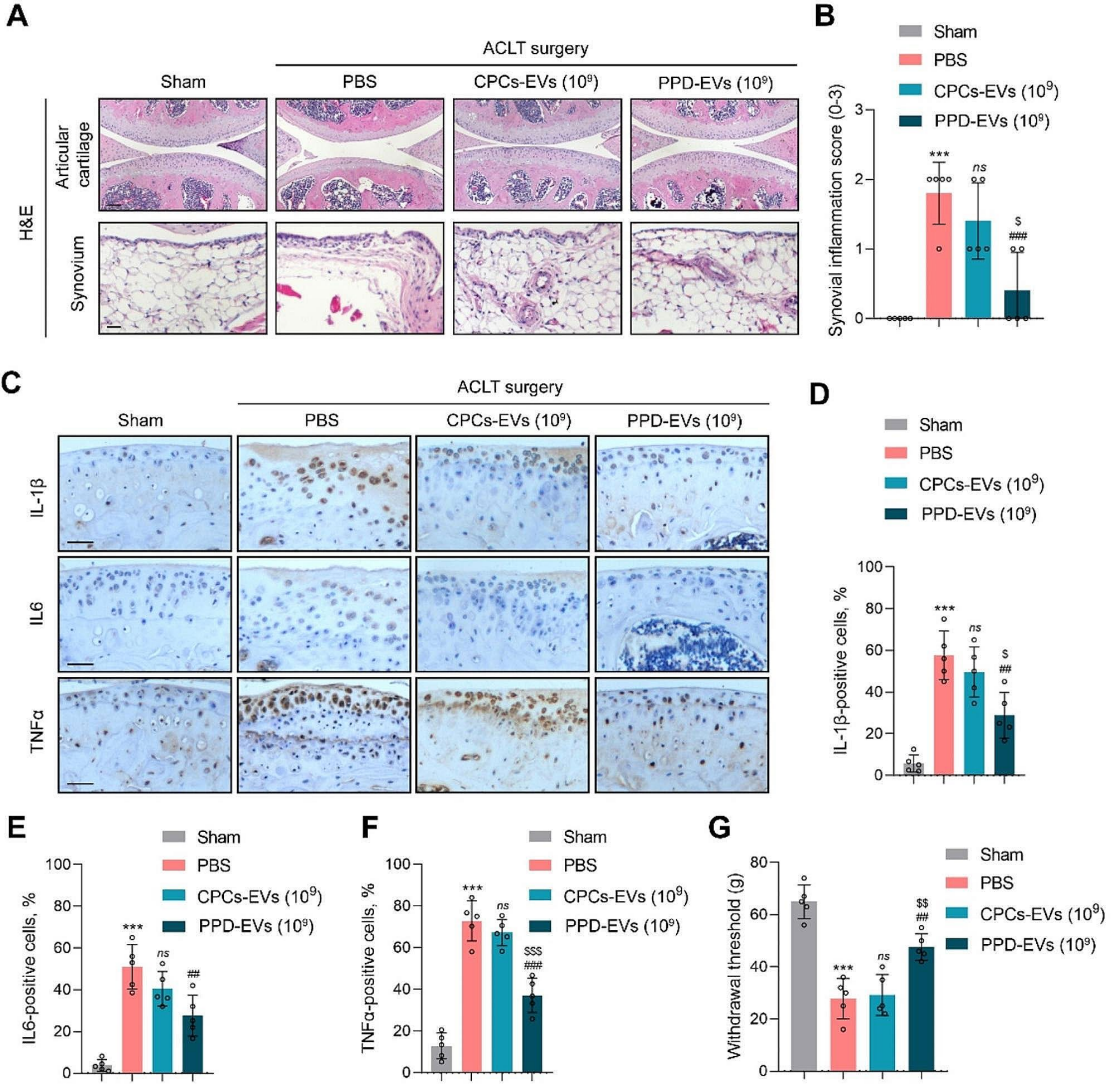
PPD-EVs ameliorate joint inflammation and OA-related pain in ACLT-induced mice. **A** Light microscopy images of articular cartilage and synovium by the H&E staining in all groups were observed and analyzed. Scale bar = 100 μm. **B** The score of synovium inflammation in each group was evaluated. **C** IHC staining for IL-1β, IL6, and TNFα in articular cartilage of mice after treatments. Scale bar = 100 μm. **D** Quantification of IL-1β-positive cells in each group. **E** Quantification of IL6-positive cells in each group. **F** Quantification of TNFα-positive cells in each group. **G** Withdrawal threshold in each group was assessed. Data represent mean ± S.D. ***P < 0.001 versus the Sham group. ^##^*P* < 0.01, ^###^*P* < 0.001 versus the HA group. ^$^*P* < 0.05, ^$$^*P* < 0.01, ^$$$^*P* < 0.001 versus the CPCs-EVs (10^9^) group. *ns*, not significant

As joint inflammation is the essential factor in the pathogenesis of OA, we further explored the regulative effect of CPCs-EVs on joint inflammation in ACLT-induced mice. H&E staining results showed that 1 × 10^9^ particles/ml PPD-EVs effectively alleviated synovitis (Fig. 6A) and the synovial inflammation score was declined from 1.80 ± 0.40 to 0.40 ± 0.49 in ACLT-induced mice, while the synovial inflammation score in the 1 × 10^9^ particles/ml CPCs-EVs-treated group showed no difference with the PBS-treated group (Fig. 6B). Moreover, IHC staining for inflammatory biomarkers in articular cartilage indicated that the expressions of IL-1β, IL6, and TNFα were effectively decreased after the treatment of 1 × 10^9^ particles/ml PPD-EVs (Fig. 6C-F). However, 1 × 10^9^ particles/ml CPCs-EVs failed in down-regulating the expressions of inflammatory markers. Furthermore, RT-qPCR analysis for IL6 and TNFα in infrapatellar fat pad also demonstrated that 1 × 10^9^ particles/ml PPD-EVs effectively decreased the gene expressions of IL6 and TNFα in mouse knee joint (Fig. S7). Next, to confirm the importance of the STAT3 pathway in the protective effects of CPCs-EVs on OA cartilage in vivo, we detected the expression of p-STAT3 in ACLT-induced mice. Consistent with in vitro findings, IHC staining results showed that intra-articular injection of PPD-EVs effectively inhibited the expression of p-STAT3 in ACLT-induced mice (Fig. S8A) and the percentage of p-STAT3-positive chondrocytes were decreased after the treatment of PPD-EVs (Fig. S8B).

Additionally, as the pain relief is an essential indicator of OA management, we further analyzed OA-related pain recovery by the hind-paw withdrawal threshold method. Compared with the PBS-treated group (27.80 ± 6.88 g), intra-articular injection of 1 × 10^9^ particles/ml PPD-EVs effectively relieved pain as withdrawal threshold was raised from 27.80 ± 6.88 g to 47.6 ± 4.50 g (Fig. 6G). However, there is no difference between PBS-treated group and 1 × 10^9^ particles/ml CPCs-EVs-treated group (Fig. 6G). Together, these results indicated that 1 × 10^9^ particles/ml PPD-EVs successfully reduced joint inflammatory response and alleviated OA-related pain in ACLT-induced mice.

#### PPD-EVs promote cartilage matrix anabolism and inhibit inflammation in cultured OA cartilage explants

**Fig. 7:**
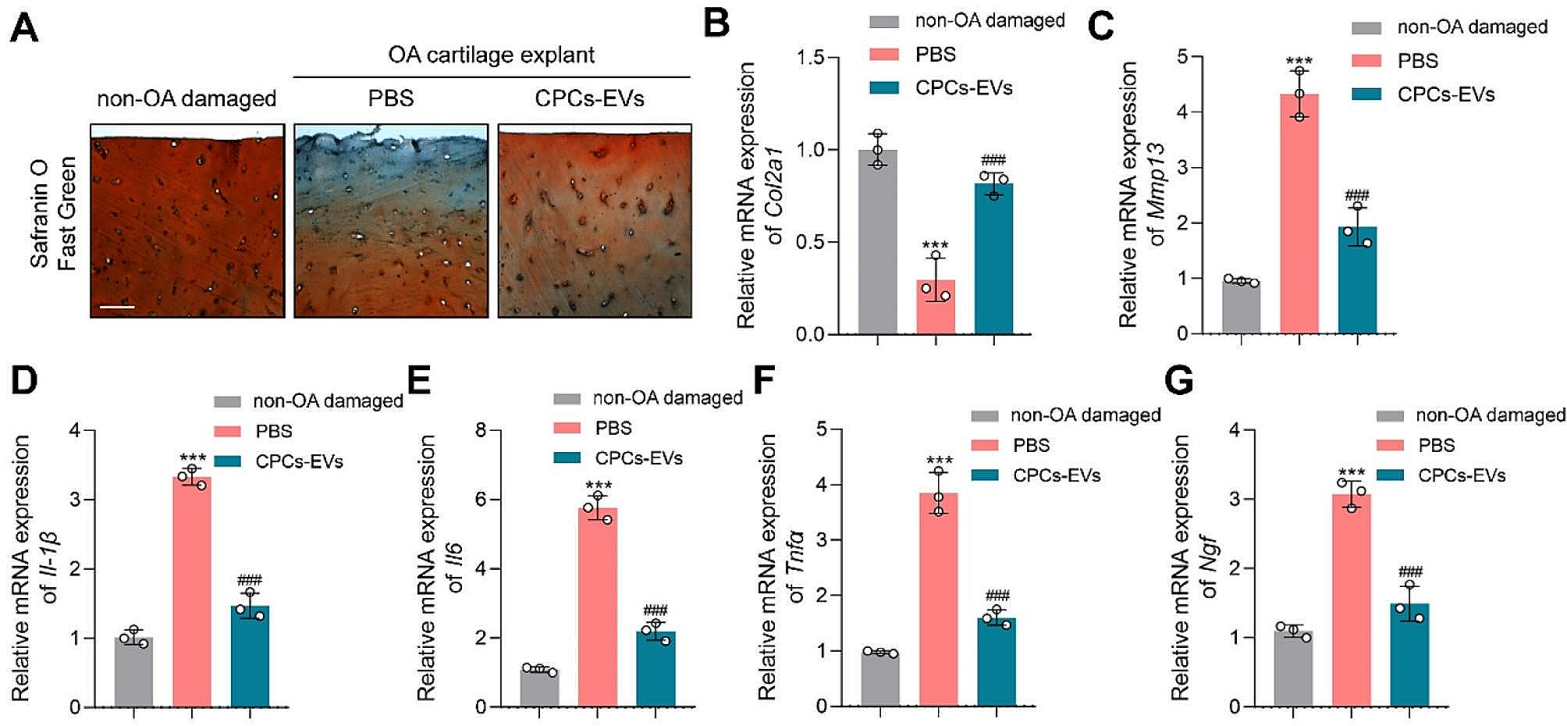
PPD-EVs alleviate cartilage destruction and inhibit inflammatory response in ex-vivo cultured OA cartilage explants. **A** Light microscopy images of articular cartilage stained by the Safranin O-Fast Green in ex-vivo cultured OA cartilage explants after treatment. Scale bar = 150 μm. **B** Relative mRNA expression of cartilage anabolism-related gene *Col2a1* was detected by PCR analysis. **C** Relative mRNA expression of cartilage catabolism-related gene *Mmp13* was detected by PCR analysis. **D-G** Relative mRNA expressions of inflammatory cytokines (*Il-1β*, *Il6*, *Tnfα*, and *Ngf*) were detected in OA cartilage explants after treatment by PCR analysis. Data represent mean ± S.D. ***P < 0.001 versus the non-OA damaged group. ^###^*P* < 0.001 versus the PBS group

Next, we further explored the relevance of our in vitro and in vivo findings to human using ex-vivo cultured human OA cartilage explants. Our results showed that PPD-EVs effectively reversed cartilage loss and promoted matrix anabolism (Fig. 7A). Consistently, PPD-EVs also promoted the mRNA expressions of anabolism genes *Col2a1* (Fig. 7B), *Acan* (Fig. S9A), and *Prg4* (Fig. S9B) in cultured human OA cartilage. In addition, PCR analysis showed PPD-EVs effectively inhibited the expressions of catabolism markers *Mmp13* (Fig. 7C) and *Adamts5* (Fig. S9C) in ex-vivo cultured human OA cartilage.

Furthermore, our results also demonstrated that PPD-EVs effectively decreased the mRNA levels inflammatory cytokines including *Il-1β*, *Il6*, *Tnfα*, and *Ngf*, confirming the inhibition of inflammatory response (Fig. 7D-G). Together, these results suggested that PPD-EVs effectively inhibited inflammatory response and maintained matrix homeostasis in ex-vivo cultured human OA cartilage explants.

## Discussion

Current treatments in clinic for OA patients include pain alleviation and total joint replacement. The former is only limited to relieving OA-related pain, and the eventual surgery is associated with intra-articular infection and other comorbidities [6, 7]. To date, there is no effective disease-modifying drug that could prevent or alleviate OA progression [51, 52].

Recently, some studies demonstrated that CPCs derived from the superficial layer of articular cartilage are promising cells for cartilage tissue engineering because of their self-proliferative capacity and strong chondrogenic differentiation ability [18, 19]. A recent study showed that the chondrogenic differentiation ability of CPCs was higher than that of bone marrow derived-MSCs, indicating that CPCs exhibit a great chondrogenic capacity [19]. Additionally, a comparative study revealed CPCs showed lower expression of the hypertrophy biomarker Collagen X in cartilage than MSCs [20]. Furthermore, previous have shown that CPCs could form stratified tissues in cartilage repair and regeneration [12, 21, 53, 54]. However, direct transplantation of stem cells has several disadvantages including the risk of immune rejection and potential tumorigenesis [55, 56]. Therefore, exploring a better strategy that can make full use of the benefits of CPCs without these potential risks is important and urgent.

Previous studies have demonstrated that MSCs-derived EVs have similar chondro-protective effects in OA cartilage as their parental MSCs. However, direct transplantation of MSCs in affected areas of articular cartilage have been shown to fabricate hypertrophy and fibrocartilage [57]. Thus, MSCs-derived EVs-based therapy is still controversial for clinic use in OA patients. Therefore, we need to explore an ideal cell source for clinic use in cartilage repair and OA treatment. A recent study indicated that CPCs-EVs can effectively promoted chondrocyte proliferation and migration in vitro [27]. This study also demonstrated that intra-articularly injection of CPCs-EVs attenuated OA progression in mice [27]. Nevertheless, the knowledge about CPCs-EVs is still limited, more studies should be performed to evaluate the chondro-protective effects of CPCs-EVs deeply and comprehensively before clinical application. Consistent with the previous study, our results showed that CPCs-EVs exhibited strong chondrogenic capacity in vitro. In IL-1β-induced chondrocytes, CPCs-EVs successfully promoted cell proliferation and enhanced the secretion of extracellular matrix. In animal studies, we further modified CPC-EVs with the cationic peptide PPD to improve bioavailability, and our results showed PPD modified CPC-EVs presented stronger cartilage penetrating ability and joint retention capacity. We also verified CPCs-EVs successfully reduced cartilage degeneration and promoted matrix anabolism in ACLT-induced posttraumatic OA mice. Additionally, our results demonstrated that PPD modified CPCs-EVs effectively maintained cartilage matrix homeostasis in ex-vivo cultured OA cartilage explants.

Chronic unresolved inflammation plays an essential role in OA progression and joint pain. Both mechanical stress and aging can lead to excessive inflammatory response in articular cartilage, which in turn activating downstream pathways, resulting in the imbalance between cartilage matrix catabolism and anabolism [58–60]. Analysis of cartilage tissues from both OA patients and OA animals has revealed that inflammatory factors have been implicated in the pathogenesis of OA [59]. Convincing evidence suggested that inhibition of inflammation effectively rescue cartilage destruction in OA animals [61, 62]. Our in vitro results showed CPCs-EVs effectively decreased the expressions of inflammatory cytokines. Our animal studies demonstrated that positively charged PPD-EVs successfully inhibited the secretion of inflammatory factors and alleviated OA-related pain in ACLT-induced mice. In ex-vivo cultured OA cartilage explants, we also found that PPD-EVs down-regulated the expressions of inflammatory factors. Moreover, our results indicated that STAT3 inhibition plays an important role in the chondro-protective effects of CPCs-EVs in OA cartilage and chondrocytes. We found 10 proteins which can regulate STAT3 pathway were encapsulated in CPCs-EVs. Consistent with our finding, a previous study has demonstrated that intra-articular injection of PPD-EVs effectively attenuated cartilage degeneration in destabilization of the medial meniscus-induced OA mice by delivering miRNA 221-3p [27] which can inhibit STAT3 signaling activation [63, 64]. Altogether, these results verified that CPCs-EVs exert chondro-protective effects at least partially by inhibiting STAT3 activation.

The current work provides proof of concept that CPCs-EVs-based therapy can be used to promote cartilage repair and regeneration in OA. However, there are still some challenges and limitations need to be overcome. First, in cartilage tissue engineering, the major concern about the clinic application of CPCs-EVs is the difficulty in obtaining large amounts of standard and functional CPCs. As the harvest of enough functional CPC-EVs is challenging and currently our research group is exploring using induced pluripotent stem cells-derived cartilage progenitor cells (iCPCs) to produce EVs (iCPC-EVs) for cartilage regeneration and OA treatment. This strategy is more efficient and could be a promising therapy for clinical application. Second, as it is not applicable to perform weekly intra-articular injection in patients with OA, optimizing the administration method of cells therapy for enhanced bioavailability should be addressed in further research. Moreover, positively charged CPCs-EVs were intra-articularly injected in the knee joint of OA mice for 6 weeks, whether it can exert chondro-protective effects in a shorter or longer therapeutic time need further research. As CPCs were cultured with 10% FBS culture medium for expansion, there is still a possibility that the internal contents of CPCs-EVs could be influenced by FBS. In addition, a deeper understanding of STAT3 pathway on the chondro-protective effects of CPCs-EVs should be explored in the future. Finally, a long-term pharmacological experiments and related validation studies in large animals should be performed before clinical translation.

## Conclusions

**Fig. 8:**
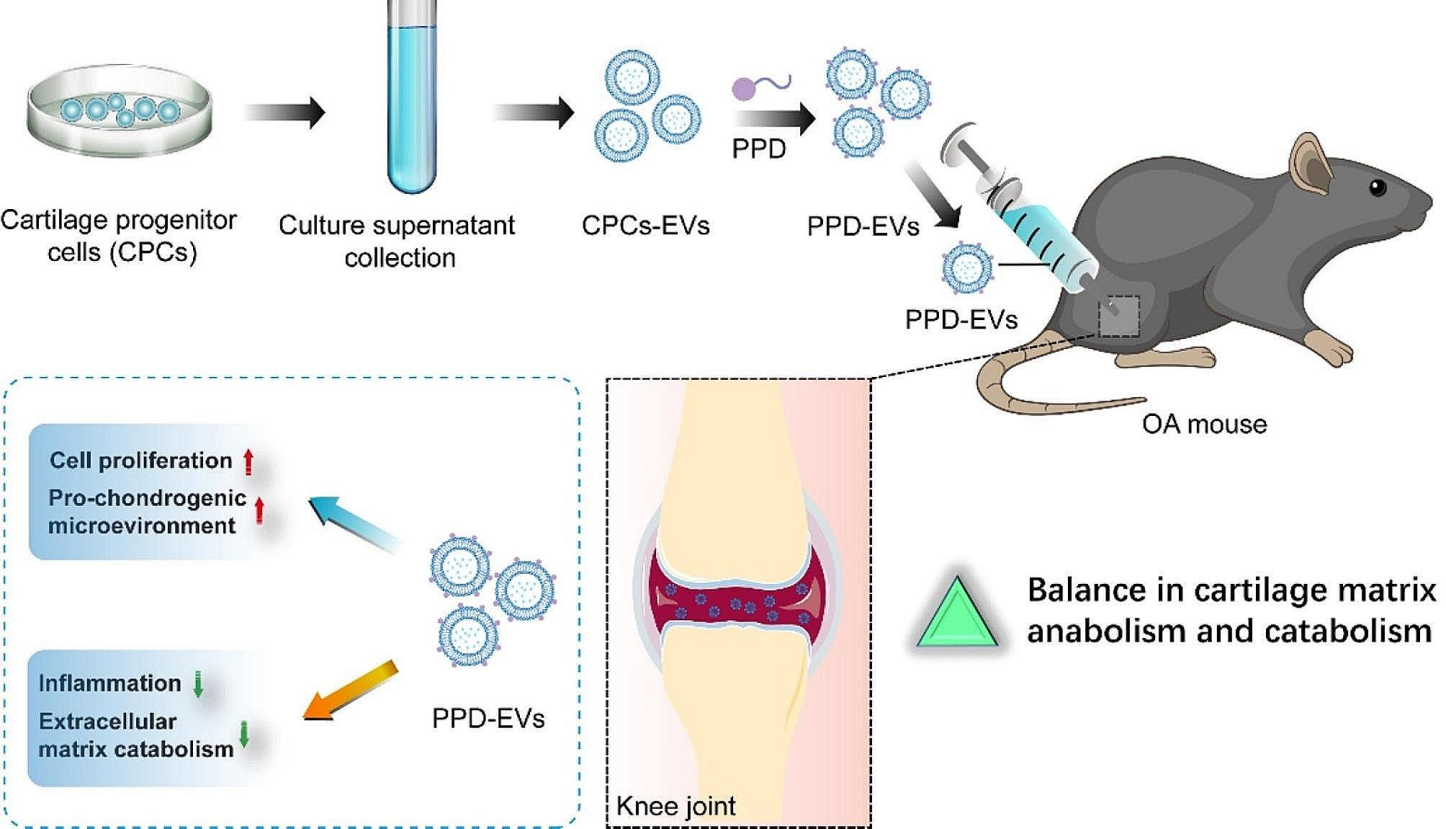
Schematic illustration of the chondro-protective effects of positively charged PPD-modified CPCs-EVs (PPD-EVs) in OA mice. Intra-articular injection of PPD-EVs promotes chondrocyte proliferation and extracellular matrix anabolism, inhibits inflammatory response and catabolism, and ultimately alleviates cartilage destruction in OA mice

Collectively, our study demonstrated that CPCs-EVs exert chondro-protective effects in vitro. Intra-articular injection of positively charged PPD-EVs more effectively maintain extracellular matrix homeostasis, alleviate cartilage degeneration, and relieve OA-related pain in ACLT-induced OA mice by inhibiting inflammatory responses (Fig. 8). Additionally, we confirmed that STAT3 inhibition plays an essential role in the chondro-protective effects of CPCs-EVs and there are 10 STAT3 regulatory proteins are encapsulated in CPCs-EVs. Collectively, CPCs-EVs-based therapy may therefore be applicable to regenerating injured cartilage tissue and is potential therapy for OA patients.

### Electronic supplementary material

Below is the link to the electronic supplementary material.

Supplementary Material 1

## Data Availability

No datasets were generated or analysed during the current study.
