# Supplementary material for: Cartilage progenitor cells derived extracellular vesicles-based cell-free strategy for osteoarthritis treatment by efficient inflammation inhibition and extracellular matrix homeostasis restoration
Source: J Nanobiotechnology. 2024 Jun 19;22:345. doi: 10.1186/s12951-024-02632-z (PMC11186174; doi:10.1186/s12951-024-02632-z)
Supplement: Supplementary file 1 — Supplementary Material 1 [file 12951_2024_2632_MOESM1_ESM.docx]

**Supplementary materials for**

**Cartilage progenitor cells derived extracellular vesicles-based cell-free strategy for osteoarthritis treatment by efficient inflammation inhibition and extracellular matrix homeostasis restoration**

**Kai Feng^1†^, Feng Wang^1†^, Hongfang Chen^2†^, Rui Zhang^1^, Jiashuo Liu^1^, Xiaodong Li^3^, Xuetao Xie^1*^, and Qinglin Kang^1*^**

^1^Department of Orthopedic Surgery, Shanghai Sixth People’s Hospital Affiliated to Shanghai Jiao Tong University School of Medicine, Shanghai 200233, China.

^2^Department of Orthopaedics, Shanghai Tenth People's Hospital, Tongji University, Shanghai 200072, China.

^3^Shanghai Key Laboratory of Orthopedic Implants, Department of Orthopedics, Ninth People’s Hospital, Shanghai Jiao Tong University School of Medicine, Shanghai 200011, China.

**^†^**Kai Feng, Feng Wang, and Hongfang Chen contributed equally to this work.

*Correspondence:

Xuetao Xie

xuetaoxie@163.com (X.X.)

Qinglin Kang

orthokang@sjtu.edu.cn (Q.K.)


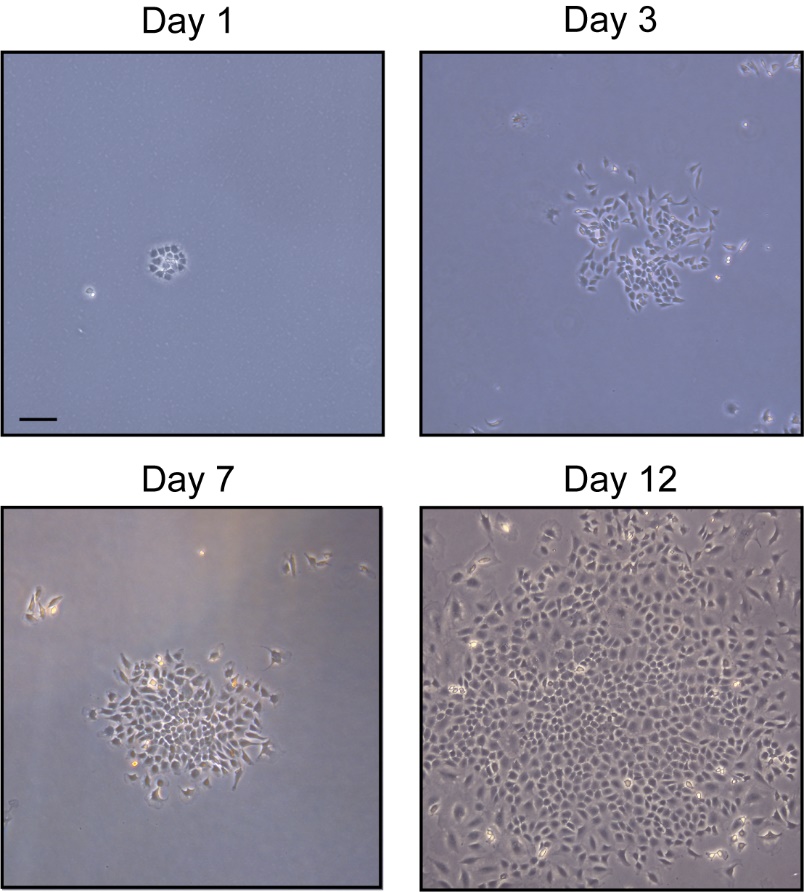


**Fig. S1** Bright field microscopy images of CPCs were captured after cultured for different days (1 day, 3 days, 7 days, and 12 days). Scale bar = 50 μm.


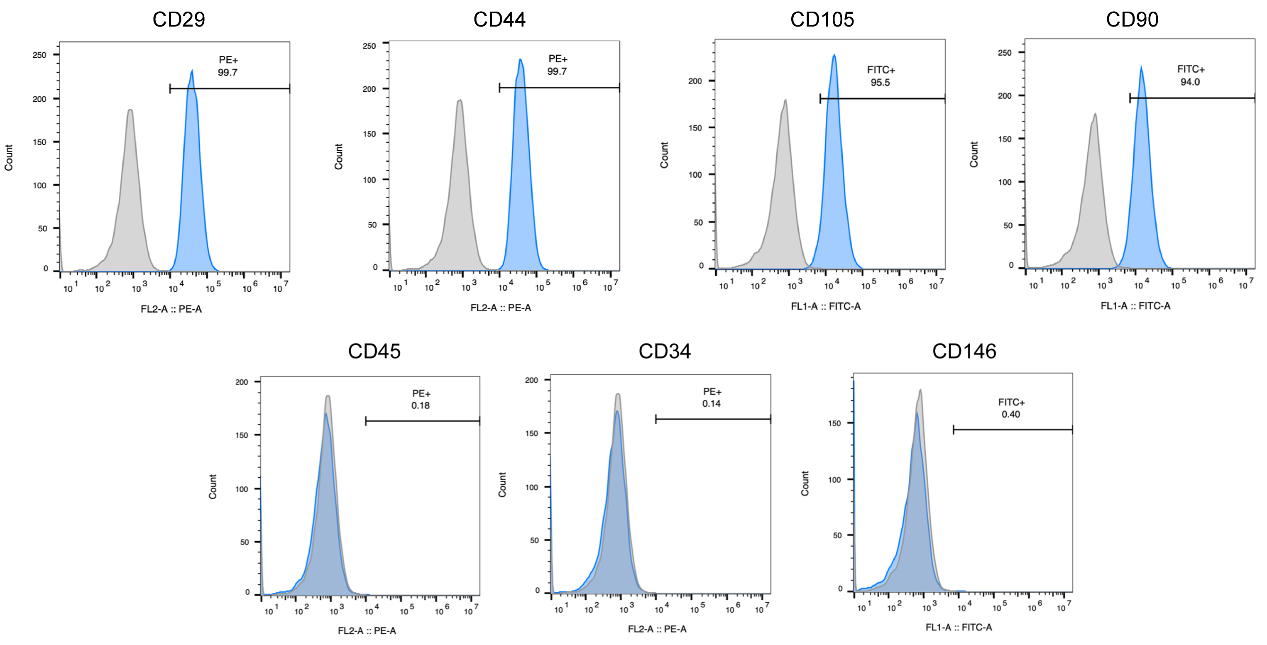


**Fig. S2** Flow cytometry analysis of CD29, CD44, CD105, CD90, CD45, CD34 and CD146 in CPCs.


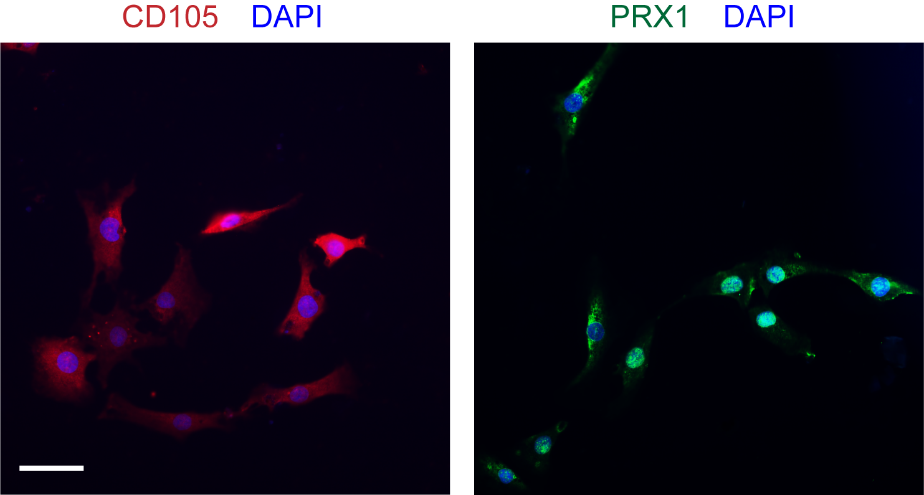


**Fig. S3** Immunofluorescence staining for CD105 (Red) and PRX1 (Green) in CPCs.


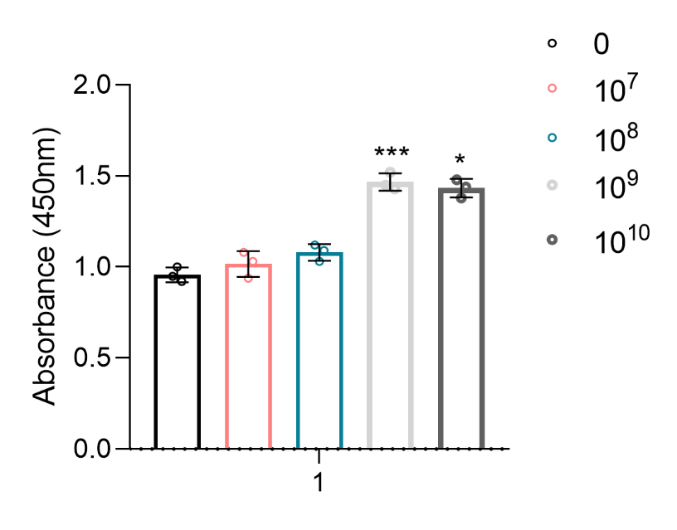


**Fig. S4** Chondrocyte proliferative ability was detected by CCK8 assay after the treatment of CPCs-EVs at different concentrations. (n = 3). Data represent mean ± S.D.. *P < 0.05, ***P < 0.001 versus the 0 group.


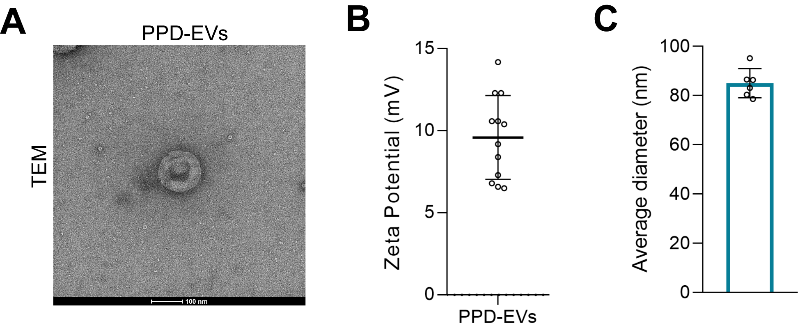


**Fig. S5** Characterization of PPD-EVs. **A** Representative morphological image of PPD-EVs captured by TEM. Scale bar = 100 nm. **B** The zeta potential of CPCs-EVs was detected by a nanoparticle analyzer. (n = 12). **C** Average particle diameter of CPCs-EVs was detected by nano-flow cytometry. (n = 6).


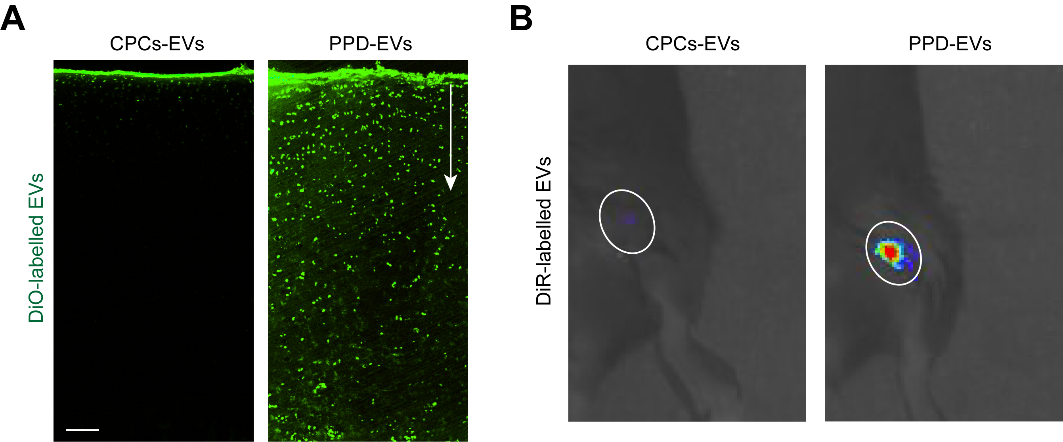


**Fig. S6** Cartilage penetration ability and joint retention capacity of CPCs-EVs and PPD-EVs.

**A** Microscope images of DiO (Green) labelled CPCs-EVs or PPD-EVs across the diffusion gradient (From up to down) of articular cartilage. Scale bar: 100 μm. **B** Representative IVIS images of mice knee joint injected with DiR-labelled CPCs-EVs or PPD-EVs for 14 days.


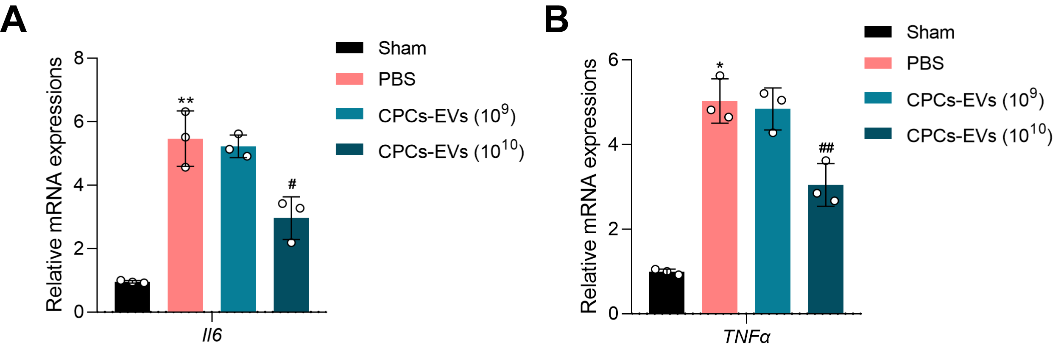


**Fig. S7** RT-qPCR analysis for inflammatory factors (*Il6* and *TNFα*) in infrapatellar fat pad of mouse knee joint. Data represent mean ± S.D.. *P < 0.05, **P < 0.01 versus the Sham group. ^#^P < 0.05, ^##^P < 0.01 versus the PBS group.


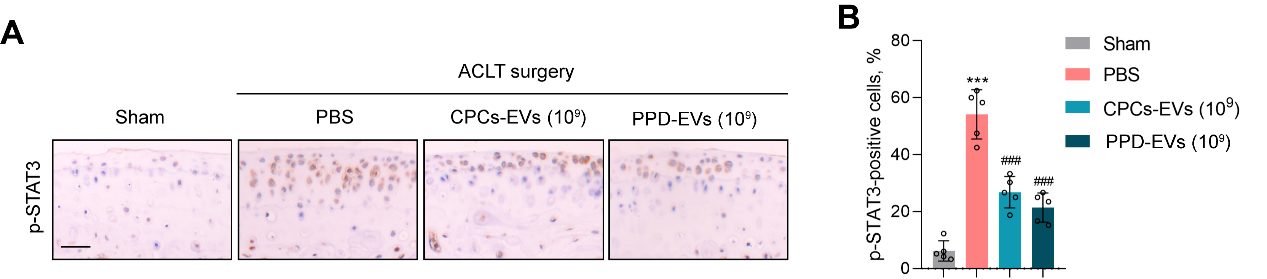


**Fig. S8** IHC staining and quantification of p-STAT3 in articular cartilage of mice after different treatments. Scale bar = 100 μm. Data represent mean ± S.D.. ^***^P < 0.001 versus the Sham group. ^###^P < 0.001 versus the PBS group.


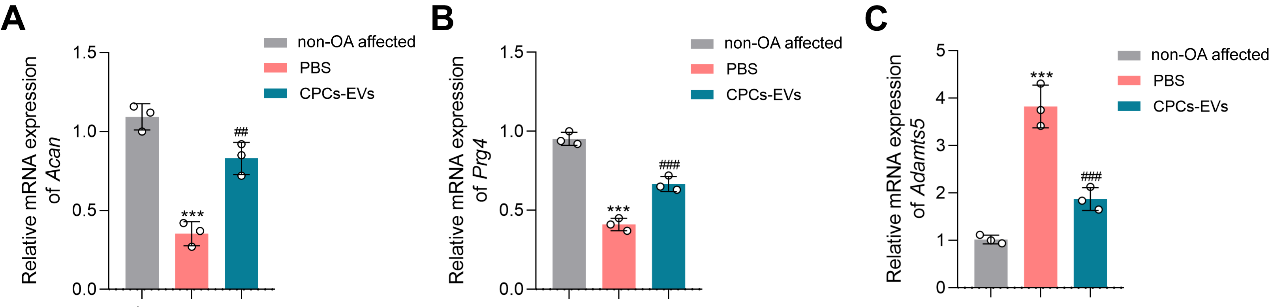


**Fig. S9** The mRNA expression of *Acan* (**A**), *Prg4* (**B**), and *Adamts5* (**C**) after PPD-EVs treatment in *ex-vivo* cultured OA cartilage explants. Data represent mean ± S.D.. ***P < 0.001 versus the non-OA damaged group. ^##^P < 0.01, ^###^P < 0.001 versus the PBS group.
